# Fear-EEG-AI: A Conceptual and Operational Framework for Designing EEG-AI Systems for the Inference of Fear-Compatible Neurophysiological States

**DOI:** 10.3390/s26196349

**Published:** 2026-10-08

**Authors:** Bladimir Serna, Ricardo Salazar, Gustavo A. Alonso-Silverio, Rosario Baltazar, Elías Ventura-Molina, Antonio Alarcón-Paredes

**Affiliations:** 1 Centro de Innovación, Competitividad y Sostenibilidad, Universidad Autónoma de Guerrero, Acapulco 39640, Guerrero, Mexico; blad.sernav@gmail.com; 2 Tecnología e Innovación (SECIHTI), Universidad Autónoma de Guerrero, Chilpancingo 39070, Guerrero, Mexico; rsalazarlo@secihti.mx; 3 Facultad de Ingeniería, Universidad Autónoma de Guerrero, Chilpancingo 39079, Guerrero, Mexico; gsilverio@uagro.mx; 4 División de Estudios de Posgrado, Tecnológico Nacional de México/Campus León, León 37290, Guanajuato, Mexico; rosario.baltazar@leon.tecnm.mx; 5 Centro de Innovación y Desarrollo Tecnológico en Computo, Instituto Politécnico Nacional, Mexico City 07738, Mexico; eventuram@ipn.mx; 6 Centro de Investigación en Computación, Instituto Politécnico Nacional, Mexico City 07738, Mexico

**Keywords:** electroencephalography (EEG), artificial intelligence, fear-compatible states, affective computing, brain–computer interfaces, explainable AI, real-world deployment, methodological framework

## Abstract

Electroencephalography (EEG) and artificial intelligence (AI) are central tools in affective computing, yet fear-related inference remains constrained by a gap between experimental performance and deployable evidence: high accuracy on controlled datasets guarantees neither neurobiological specificity, nor robustness across users, nor traceable inference. EEG does not measure fear directly; it captures cortical dynamics compatible with threat processing, arousal or vigilance. This paper introduces the Fear-EEG-AIFramework, a technology-independent architecture for designing, validating and deploying EEG–AI systems targeting fear-compatible neurophysiological states. Building on a prior systematic review, it organizes development into three hierarchical levels—neurobiological foundation, neuroelectric correlates and computational modeling—governed by six cross-cutting principles, and makes them evaluable through eight observable dimensions, the Fear-EEG-AI Alignment Index (FAI) and five validation evidence levels. Applied to the eight eligible studies of that corpus by two raters who had not drafted the instrument, with adjudication by a third, the rubric reached 67.2% exact agreement (quadratic-weighted κ=0.741; ICC(2,1) =0.744). Under 10,000 admissible weightings the equal-weight ordering survived in 10.1% of draws, and one of 56 ordered pairs met a stability criterion fixed before the pairwise results were tabulated. The index is therefore reported as a descriptor of methodological documentation and not as a ranking of implementations.

## 1. Introduction

Fear constitutes an adaptive response that is essential for human survival, coordinating physiological, cognitive, and behavioral mechanisms aimed at detecting and coping with real or perceived threats [1,2]. When this response arises in contexts of vulnerability, isolation, or limited supervision, however, its early identification can acquire a practical relevance that extends beyond purely neuroscientific interest. In older adults who live alone, for instance, episodes of fear associated with falls, disorientation, unexpected medical events, or acute anxiety may substantially compromise physical and psychological well-being [3]. Despite advances in remote assistance and intelligent monitoring systems, many solutions continue to prioritize observable physical variables—such as mobility, activity, or fall detection—while emotional and neurophysiological states remain less integrated into preventive care schemes.

Against this backdrop, affective neuroscience and affective computing have opened the possibility of studying emotional states from objective physiological signals [4]. Among the available technologies, electroencephalography (EEG) holds a particularly relevant position owing to its non-invasive nature, its growing portability, and its high temporal resolution for capturing the neuroelectric dynamics associated with emotional processes [5]. In parallel, artificial intelligence techniques have expanded the capacity to analyze complex patterns in EEG signals, enabling models designed to infer affective and cognitive states from computational representations of brain activity [6,7].

Physiological affect recognition is not confined to a single modality, and much recent work is explicitly multimodal. Peripheral autonomic activity can be recovered with optical-fiber cardiorespiratory sensing and facial thermal signatures with infrared imaging, and the two have been combined for multimodal emotion recognition [8]; infrared thermography has been used on its own to index affective states from facial emissivity [9]; and deep representation learning has been applied to emotion recognition from heterogeneous physiological channels [10]. These approaches are complementary to the one addressed here rather than alternatives to it, and the difference matters for the argument that follows. Peripheral and thermal channels index the autonomic and vascular consequences of affective processing, which are shared across negatively valenced states of high arousal; EEG is the only one of these modalities that records cortical activity directly, and therefore the only one in which the relationship between a measured correlate and a specific affective construct can be posed as a neurophysiological question at all. That is also why the alignment problem this work addresses is sharper for EEG than for peripheral modalities: the closer a signal is to the process of interest, the less excusable it becomes to leave the link between construct and correlate unstated. The framework proposed here is accordingly unimodal by design, not by omission, and Section 4.5 states what that scope excludes.

The convergence of EEG and artificial intelligence has yielded promising results in emotion recognition, brain–computer interfaces, and neuroadaptive systems. The literature reports numerous combinations of biomarkers, feature-extraction strategies, and computational models with seemingly high performance in classifying or recognizing emotional states [11,12]. These advances, however, are accompanied by considerable methodological heterogeneity. Studies differ in their experimental protocols, EEG acquisition setups, preprocessing criteria, validation schemes, algorithmic architectures, and reporting practices. Although this diversity reflects a dynamic field, it also hampers cross-study comparison, limits reproducibility, and constrains the translation of experimental findings into cumulative design criteria.

The gap becomes even more critical when the goal is not merely to classify signals under controlled conditions but to advance toward EEG–AI systems capable of operating in real-world settings. In the fields of brain–computer interfaces and applied neurotechnology, it has been repeatedly noted that laboratory performance does not guarantee equivalent behavior during everyday use [13,14]. Factors such as inter-subject variability, contextual change, the presence of artifacts, the temporal stability of signals, computational constraints, energy requirements, and the need for continuous operation introduce challenges that are not always considered from the earliest design stages. As a result, many developments remain experimental demonstrations with limited readiness for deployment.

A recent systematic review on the identification of fear-compatible neurophysiological states using EEG and artificial intelligence synthesized these limitations and showed that the problem does not stem solely from a lack of available algorithms, devices, or biomarkers [15]. Rather, the evidence points to the need for methodological structures capable of articulating—within a single design rationale—the neurobiological foundations of fear, its observable neuroelectric correlates, computational modeling strategies, validation criteria, and the constraints inherent to deployment. In the absence of such a structure, the development of EEG–AI systems risks fragmenting into isolated technical decisions that are difficult to compare, justify, or transfer to real application contexts.

In response to this need, the present work proposes the Fear-EEG-AI Framework, a conceptual and methodological architecture intended to guide the design, evaluation, and eventual deployment of EEG–AI systems for identifying fear-compatible neurophysiological states. The framework does not seek to prescribe a specific EEG device, biomarker, or algorithm. Its purpose is to provide an organizing pathway that connects neuroscientific, neuroelectric, computational, and operational decisions under a shared structure. To this end, the proposal is organized into three complementary levels: neurobiological foundations, neuroelectric correlates, and computational modeling. Spanning these levels are cross-cutting principles concerning neurophysiological coherence, technological independence, deployment-oriented design, inference traceability, validation under progressively realistic conditions, and operational feasibility.

The main contribution of this work is therefore to formulate a conceptual–operational framework for organizing the development of EEG–AI systems targeting fear-compatible states, rather than to introduce a new biomarker, algorithm, or device. Three differences delimit that contribution. With respect to the authors’ earlier systematic review [15], which supplies the study corpus used below but none of the structure proposed here, the present work moves from a descriptive synthesis of reported practice to an operational instrument whose measurement properties are characterized. With respect to construct-agnostic reporting guidance such as TRIPOD+AI and PROBAST+AI [16,17], it adds the layer those instruments do not address: whether the inferred affective state is delimited from the adjacent states that share its electrophysiological signature. With respect to readiness scales such as TRL and SRL [18,19], it shifts the object of description from the maturity of an artifact to the type of empirical evidence that supports an inferential claim. Specifically, the manuscript: (i) structures the neurobiological, neuroelectric, and computational components relevant to this domain; (ii) defines design principles that support the transition from experimental research toward potentially deployable applications; (iii) proposes observable dimensions for reporting and evaluating implementations derived from the framework; and (iv) applies the framework and its instruments to a defined corpus of published studies and characterizes their behavior. From this perspective, the Fear-EEG-AI Framework seeks to help narrow the gap between the available experimental evidence and the development of EEG–AI systems that are more robust, traceable, reproducible, and ready for real-world application.

### RelatedReporting and Readiness Frameworks

The concern that motivates this work—that predictive performance alone cannot establish whether a system is sound, reproducible, or transferable—is not specific to EEG. It has produced a family of reporting and readiness instruments against which any new framework in this space must be positioned. Four groups are relevant here.

The first group standardizes the reporting of predictive and clinical artificial intelligence: TRIPOD+AI and PROBAST+AI for the reporting and risk-of-bias appraisal of prediction models, CONSORT-AI and SPIRIT-AI for trials and trial protocols, and DECIDE-AI for early-stage clinical evaluation [16,17,20,21,22]. All are agnostic to the signal modality and to the target construct: they ask whether a model was developed, validated and reported transparently, not whether the quantity it predicts is neurophysiologically identifiable.

The second group standardizes electrophysiological practice: the COBIDAS MEEG recommendations and the EEG-BIDS specification define what must be reported and how data must be organized for EEG research to be reproducible [23,24], in far greater technical detail than this framework, so dimension D3 below adopts rather than replaces them. The third describes technological maturity: Technology Readiness Levels and Systems Readiness Levels characterize the progression of an artifact from concept to operational use [18,19], but not the type of empirical evidence supporting an inferential claim about a latent affective state. The fourth is regulatory: Regulation (EU) 2024/1689 restricts emotion-recognition systems in workplace and educational contexts and classifies several affective-inference applications as high risk [25], delimiting which deployments are admissible at all.

Table 1 summarizes this landscape and identifies what remains uncovered. None of these instruments addresses whether the target affective construct is delimited from adjacent states that share its physiological signature, whether the chosen biomarkers are defensible as correlates of that construct rather than of generic arousal, or the interaction between the deployment constraints of consumer-grade EEG and the signal quality the inference requires. Those three gaps define the contribution of the present work, which stands as a domain specialization inheriting from these frameworks rather than an alternative to them. A crosswalk between the eight dimensions of Section 3.2 and the principal items of the TRIPOD+AI checklist is provided as Supplementary Materials; it records, for each dimension, the items that already cover it and what remains uncovered. One entry of that mapping is worth stating here: dimension D4 is covered by the existing checklist essentially in full, and is therefore best read as a pointer to it rather than as a contribution of this framework. Alignment with COBIDAS MEEG and EEG-BIDS is handled differently, by adopting their recommendations within dimension D3 rather than by restating them.

## 2. Conceptual Architecture of the Fear-EEG-AI Framework

The Fear-EEG-AI Framework is conceived as a conceptual and methodological architecture for organizing the design of EEG–AI systems aimed at identifying fear-compatible neurophysiological states. Its purpose is not to propose a single model, a closed algorithmic architecture, or a specific technological platform. Rather, it seeks to provide a modular structure that allows design decisions to be justified across three complementary planes: the neurobiological basis of the phenomenon under study, the neuroelectric correlates that can be observed through EEG, and the computational strategies used to transform those signals into interpretable inferences.

The rationale of the framework responds to the need to avoid two methodological extremes. On the one hand, purely algorithmic approaches may achieve high performance without clearly explaining which neurophysiological aspects underpin the inference. On the other hand, exclusively theoretical models may prove of little operational value if they are not translated into observable, processable, and measurable variables. Accordingly, the framework adopts a “grey-box” perspective [26]: computational decisions must be anchored in neuroscientific foundations, while neurobiological constructs must be expressed through indicators amenable to measurement, modeling, and validation.

This architecture is derived from the findings synthesized in the systematic review on the identification of fear-compatible states using EEG and artificial intelligence [15]. That evidence showed that the available literature contains heterogeneous biomarkers, algorithms, and experimental protocols but lacks a common methodological pathway linking neurobiological validity, computational traceability, and deployment feasibility. Building on this gap, the Fear-EEG-AI Framework is organized into three hierarchical and interdependent levels: (i) the neurobiological foundation, (ii) the neuroelectric correlates of fear, and (iii) computational modeling. These levels do not represent isolated stages but rather layers of decision-making that must remain coherent throughout the entire system-design cycle.

### 2.1. Level 1: Neurobiological Foundation

Any EEG–AI system aimed at fear-compatible states must begin by delimiting the neurobiological construct it intends to approximate. This delimitation is necessary because EEG does not measure emotions directly; rather, it captures variations in cortical electrical activity that may, under certain conditions, be associated with affective, cognitive, and physiological processes. A “black-box” approach that feeds raw EEG data to a classifier without an explicit neurophysiological hypothesis may learn artifacts, protocol-induced biases, or spurious correlations instead of patterns related to the phenomenon of interest [7]. Level 1 therefore establishes that the initial objective is not to “detect fear” as an absolute psychological category, but to define which components of the brain circuitry associated with threat response can produce effects observable through EEG.

Research in affective neuroscience has identified a central—though not exclusive—circuit involved in the appraisal of and response to threats [27]:

- Amygdala: involved in the rapid appraisal of potentially threatening stimuli and in assigning emotional salience to environmental cues [1].
- Prefrontal cortex (PFC): involved in the regulation, contextual interpretation, and cognitive modulation of the fear response, particularly when the individual must distinguish between a real threat, a perceived threat, and an emotionally intense but safe stimulus.
- Insula: contributes to interoceptive processing—that is, the perception of the body’s internal state, such as increased heart rate, tension, or physiological arousal—components relevant to the subjective experience of fear [28].

This formulation introduces a key constraint for the design of EEG–AI systems. Scalp EEG, especially when acquired with portable, low-cost, or low-channel-count devices, does not allow the precise localization of activity in deep structures such as the amygdala. It can, however, register the cortical consequences associated with the activation and modulation of networks involved in threat response. In particular, the functional interaction between limbic structures and prefrontal regions makes it reasonable to assume that part of the emotional dynamics may be expressed as changes in cortical oscillations accessible to EEG sensors [29]. From this perspective, amygdala-related activity is not interpreted as a signal directly measurable by EEG, but as a process that can modulate cortical patterns, particularly in frontal and prefrontal regions [27].

Naming that modulation as a mechanism, rather than leaving it as an assumption, narrows what a system can claim. Cross-frequency coupling is the candidate most often proposed for communication between anatomically distant populations oscillating at different rates, with the phase of a slower rhythm organizing the amplitude of a faster one [30]; in the threat domain, functional coupling between the amygdala and dorsomedial prefrontal cortex has been reported during the processing of threat-related material [31]. Frontocentral theta–gamma phase–amplitude coupling is therefore a plausible surface-recordable correlate of limbic–prefrontal communication, and frontal midline theta is the component of that pair for which a functional interpretation is best established [32]. Two qualifications apply and both are part of the framework rather than caveats to it. Coupling estimates are sensitive to epoch length, filter settings and amplitude-phase dependencies, so they are not interchangeable across acquisition configurations; and no coupling measure has been established as specific to threat processing as opposed to other demanding affective or cognitive states. On that basis the mechanism is admissible as a rationale for looking at frontocentral activity, and not as evidence that a given system measures fear.

Level 1 thus yields a fundamental design conclusion: the objective of the framework is neither to measure the amygdala directly nor to claim absolute detection of fear, but to approximate fear-compatible neurophysiological states from observable cortical effects that are theoretically linked to threat-appraisal circuits. This conceptual precision helps avoid overstated claims and provides a more solid basis for selecting EEG biomarkers at Level 2.

#### Delimitingthe Fear-Compatible State

The construct this framework targets must be delimited before any biomarker is selected, because the states from which it must be distinguished share most of its known electrophysiological signature. The target construct is accordingly defined both positively and negatively.

Positively, a fear-compatible neurophysiological state is a transient cortical configuration, elicited by a stimulus or context appraised as threatening, that is consistent with the engagement of threat-appraisal and defensive-response circuitry, and that is expressed at the scalp as a coordinated change across several frequency bands over frontal and frontotemporal regions.

Negatively, the construct is not the conscious experience of fear, is not identifiable with a single oscillatory marker, and is not separable, on EEG evidence alone, from anticipatory anxiety, acute stress, sustained vigilance, high cognitive load, startle, or negative valence at high arousal. This is why the framework retains the qualifier compatible with throughout: the inference licensed by the evidence is one of consistency with a class of states, not identification of one member of that class. The distinction between defensive survival circuits and the conscious experience that may accompany them is well established in the affective neuroscience literature [33,34], as is the absence of consistent, state-specific physiological signatures for discrete emotion categories [35].

Table 2 makes this confusion space explicit. Each row names a state the target construct must be distinguished from, the paradigm that typically elicits it, the EEG correlates reported for it, and the evidence that would be required to discriminate it from the target construct. The overlap column is, with one partial exception, uniformly high. Declaring that overlap is a requirement of the framework rather than a concession: an implementation that reports the confusion space it considered, and the evidence it has for excluding its members, is making a falsifiable claim; one that reports an accuracy figure for a label called “fear” is not.

Two consequences follow. First, the correlates described in Section 2.2 are to be read as candidate correlates, none of which is specific to the construct, and whose joint configuration—not any single member—carries the inferential weight. Second, the highest anchor of D1 is reserved for studies that state their confusion space explicitly; on the present evidence base that is a demanding rather than a routine requirement.

The distinctions in Table 2 are conceptual, and the framework does not claim that they are resolvable from scalp recordings: no electrophysiological signature is known that isolates threat appraisal from generalized sympathetic arousal, and Section 2.2 states why the correlates available do not provide one. What the framework requires instead is that a study claiming to have discriminated the target state from an adjacent one make that claim evaluable. Three conditions are necessary for it to be so.

First, the comparison must include at least one adjacent state of comparable arousal, and not only a neutral or resting condition. A model that separates threat-related material from a neutral baseline has established nothing about threat versus intense anger, and the two claims are routinely conflated. Second, the matching of arousal across conditions must be reported—by self-report, by a peripheral measure, or by normative stimulus values—so that an observed difference is not attributable to arousal level alone. Third, the states of Table 2 that were not contrasted must be named. An implementation that discriminates its target from two adjacent states and declares the remaining ones untested is more informative than one reporting a single accuracy figure against a neutral condition.

These are reporting conditions on a discrimination claim, not criteria that confer specificity. Stating them is what allows the delimitation of this section to constrain the evaluation in Section 3.9, where the absence of an adjacent-state contrast is the most frequent reason a study cannot support the interpretation it offers.

### 2.2. Level 2: Neuroelectric Correlates of Fear

The second level translates the neurobiological foundation of Level 1 into electrophysiological indicators observable through EEG. This translation is a critical stage, as it determines which aspects of the signal will be considered relevant for approximating fear-compatible neurophysiological states and which must be interpreted with caution owing to their low specificity, susceptibility to artifacts, or dependence on the experimental context.

Unlike approaches that seek a single, universal electroencephalographic signature of fear, the framework adopts an integrative neuroelectric perspective. Current evidence suggests that emotions emerge from dynamic interactions among distributed neural networks and that their EEG manifestations are typically expressed through complex oscillatory patterns involving several frequency bands [5,11]. Consequently, fear is not operationalized here as an isolated biomarker but as a configuration of complementary indicators whose interpretation must remain tied to neurobiological theory, the experimental context, and signal quality.

Several studies in affective neuroscience have reported that exposure to emotionally relevant stimuli modifies oscillatory activity in the theta (4–8 Hz), alpha (8–12 Hz), and beta (13–30 Hz) bands, particularly over frontal and frontotemporal regions [29,36]. These variations can be related to attention, emotion regulation, vigilance, cognitive appraisal of threats, and behavioral preparation. Level 2 therefore does not seek to select a single band as a definitive marker, but to establish a reasoned set of neuroelectric correlates that can guide feature extraction in EEG–AI systems.

#### 2.2.1. Alpha-Band Suppression as an Indicator of Cortical Activation

A decrease in alpha power is one of the most widely used indicators of cortical activation during the processing of emotionally significant stimuli. Alpha desynchronization is commonly interpreted as a transition from states of rest or functional inhibition toward states of increased information processing [37].

In studies of emotional processing, stimuli associated with fear, threat, or negative emotions have shown reductions in alpha activity, particularly over frontal and parietal regions [11,36]. Within the framework, alpha suppression is treated as a reference biomarker—not because it is specific to fear, but because it provides a relatively interpretable and reproducible signal that is compatible with EEG configurations of varying complexity.

Alpha power also decreases with attentional engagement, task difficulty and general arousal irrespective of affective content, and the underlying event-related desynchronization (ERD) and synchronization (ERS) dynamics are not specific to threat. Within the framework, alpha suppression is therefore a component of a configuration and is not interpretable, on its own, as evidence of a fear-compatible state.

#### 2.2.2. Beta Activity and Threat Processing

Beta activity has been associated with vigilance, sustained attention, active cognitive processing, and behavioral preparation [29]. In emotional-processing tasks, relative increases in beta power may be interpreted as signs of heightened cognitive appraisal of potentially threatening stimuli or as the involvement of regulatory mechanisms associated with frontal regions.

The framework treats the beta band as a relevant indicator because it can provide information about states of activation, alertness, and active processing. Its interpretation, however, must be carried out jointly with other biomarkers, since beta activity may also reflect motor processes, muscle tension, or cognitive demands that are not specific to fear.

Because relative beta increases also accompany motor preparation, muscle tension and general cognitive demand, the band carries information about activation rather than about threat appraisal specifically; its use presupposes that the design separates these sources.

#### 2.2.3. Theta Activity and Emotion Regulation

The theta band has been linked to mechanisms of cognitive control, the integration of emotional information, working memory, and functional communication between cortical and subcortical structures [29].

Although theta activity is not a fear-specific marker, several studies have documented frontal modulations during emotional-processing tasks [11]. The framework therefore treats it as a complementary source of information, particularly useful when the aim is to represent processes of regulation, contextual appraisal, or affective–cognitive integration within computational models.

Frontal midline theta has been associated with anxiety and with cognitive control more broadly [32], which places it among the correlates shared with the adjacent states of Table 2 rather than among discriminating ones.

#### 2.2.4. Frontal Asymmetry as a Functional Biomarker

Beyond band-wise spectral measures, the framework incorporates frontal asymmetry as a functional biomarker. The affective neuroscience literature has documented associations between differences in left- and right-prefrontal cortical activity and various emotional states [38,39].

In the context of fear, frontal asymmetry is relevant because it can capture patterns related to approach, avoidance, emotion regulation, and threat appraisal. Accordingly, when the hardware configuration includes sufficient bilateral frontal channels, asymmetry-based features should be considered priority components of the feature-extraction process.

Two qualifications apply. Frontal alpha asymmetry indexes approach–withdrawal motivation rather than threat appraisal as such, and its psychometric reliability is sensitive to reference scheme, montage and session, which limits its use as a standalone biomarker in portable configurations. It is retained here as a candidate feature whose inclusion must be justified against the montage actually available.

#### 2.2.5. Spectral Complexity and Inter-Band Ratios

Beyond band-wise power, two further families of indicators are used in the framework and are introduced here for completeness, since both are referenced in the feature-representation decisions of Level 3.

Spectral and signal complexity. Spectral entropy, differential entropy, sample entropy and fractal-dimension estimates quantify the dynamic organization of the signal. Their appeal in affective applications is that they are sensitive to changes in the temporal structure of activity that band-power measures average out, and several of them are inexpensive enough for on-device computation. Their limitation is interpretive: a change in entropy does not localise to a band or to a mechanism, so these features contribute to discriminative performance more readily than to the explanatory chain the framework requires.

Inter-band ratios. Ratios such as θ/β and α/β normalize one band against another within the same channel. Their practical value is robustness: because both terms are affected similarly by electrode impedance, inter-individual differences in skull conductance and overall signal scaling, the ratio is more stable across subjects and sessions than either absolute power alone. Their cost is that a given ratio value is not uniquely determined—numerator and denominator changes are not distinguishable—so ratios should be reported alongside the component powers rather than instead of them.

Both families are therefore treated as admissible and, in portable configurations, often advantageous representations, subject to the same requirement as all other correlates: that their relationship to the construct defined in Section 2.1 be stated rather than assumed.

#### 2.2.6. Gamma Activity as a Complementary Biomarker

Gamma activity, generally considered to lie above 30 Hz, has been associated with perceptual integration, high-level cognitive processing, and emotional response [29]. This band, however, poses an important limitation for deployable EEG–AI applications: it is especially susceptible to contamination by electromyographic artifacts produced by facial muscles, ocular movements, and jaw tension [40].

For this reason, the Fear-EEG-AI Framework does not treat gamma activity as a mandatory requirement. Instead, it is regarded as an optional feature that should be incorporated only when signal quality, sampling rate, channel count, and artifact-removal procedures are sufficient to justify its physiological reliability.

That condition is operational only if what counts as sufficient is stated, and the question is sharpest in exactly the configurations the framework targets: portable headbands place frontal electrodes over facial musculature, so the contaminating source sits closer to the sensor than the neural source of interest. The framework does not propose a signal-to-noise threshold above which gamma becomes admissible, and the reason is substantive rather than cautionary: the quantity that would have to exceed such a threshold is not comparable across montages, electrode types, reference schemes and tasks, so a single published cut-off would be applied where it does not hold and would give the appearance of control without providing it. What the framework requires instead is that the criterion actually used be reported and auditable, and it names three diagnostics that make such a criterion checkable.

The first is the aperiodic component of the power spectrum. Muscle activity broadens and flattens the high-frequency portion of the spectrum, so the exponent of the aperiodic fit is a more informative indicator of contamination than gamma power itself, which conflates periodic activity with a shifted broadband background [41]. Separating periodic from aperiodic components before reading band power is a precondition for interpreting a gamma effect, not a refinement of it. The second is spatial: electromyographic activity has a characteristic topography, concentrated at electrodes adjacent to temporalis and frontalis musculature, and its consistency across channels differs from that of cortical sources [40]. In single-channel or two-channel configurations this diagnostic is unavailable, which is itself a reason to treat gamma as inadmissible there. The third is procedural: the criterion must be justified for the montage in use and reported with the acquisition parameters, rather than inherited from a study using different hardware.

Accordingly, a study that derives an inference from correlates above 30 Hz without reporting such a criterion is treated by the assessment scheme of Section 3.2 as not having established the reliability of those correlates, irrespective of the classification performance obtained. The corresponding scoring rule is stated in Appendix A.2.

#### 2.2.7. Implications for Portable EEG Systems

The main engineering implication derived from Level 2 is that approximating fear-compatible states does not necessarily require high-density clinical EEG systems. The available evidence suggests that reduced configurations—particularly those with frontal or frontotemporal channels—can capture information relevant to emotional analysis when combined with robust signal-processing and feature-extraction strategies [5,11].

The framework therefore prioritizes biomarkers that simultaneously satisfy three criteria:

1. Neurobiological relevance to fear processing.
2. Reproducibility across different experimental paradigms.
3. Feasibility of implementation on portable, low-cost EEG devices.

This prioritization maintains a balance between neurophysiological validity and technical feasibility. Level 2 thus acts as a bridge between the fear-circuit theory described in Level 1 and the computational-representation decisions developed in Level 3.

From this perspective, alpha suppression, beta and theta modulations, frontal asymmetry, spectral-complexity metrics, and indicators derived from inter-band relationships constitute the set of candidate neuroelectric correlates considered by the Fear-EEG-AI Framework, none of which is specific to the target construct, whereas gamma activity is regarded as a complementary feature dependent on the technological context of implementation.

### 2.3. Level 3: Computational Modeling

Level 3 establishes the computational criteria required to transform the neuroelectric correlates of Level 2 into automated inferences about fear-compatible neurophysiological states. This layer should not be understood as an algorithmic prescription, but as a decision space in which the extracted features, the selected models, and the inference conditions must remain coherent with the previously defined neurobiological construct.

Unlike approaches centered on a specific technique, this level adopts a technology-independent perspective. This allows different methods of artificial intelligence, machine learning, and edge computing to be incorporated as the available tools evolve. Under this philosophy, the framework does not prescribe a single classifier but rather criteria for balancing predictive performance, interpretability, reproducibility, computational efficiency, and deployment feasibility [7,14].

Level 3 is organized around three complementary decisions: feature representation, computational-model selection, and inference strategy.

#### 2.3.1. Feature Representation

The EEG signal is an indirect, dynamic, and highly variable representation of brain activity. Therefore, before any inference model is applied, the signal must be transformed into features that capture relevant neurophysiological patterns while reducing the influence of noise, artifacts, or variability unrelated to the phenomenon of interest [7,11].

The framework proposes a multimodal representation based on four main feature categories:

1. **Spectral features.** These include measures derived from the energy distribution of the signal across frequency bands. Among the most widely used are power spectral density (PSD), absolute power, relative power, and inter-band ratios. Such features quantify phenomena such as alpha suppression, theta modulation, and the relative changes in beta activity associated with emotional states [37,42].
2. **Temporal features.** These describe statistical properties of the signal in the time domain, including mean, variance, energy, mobility, and complexity. Notable within this category are the Hjorth parameters, widely used in EEG applications owing to their low computational cost and ease of interpretation [43].
3. **Time–frequency features.** These capture the non-stationary nature of EEG through techniques such as the continuous wavelet transform (CWT), the discrete wavelet transform (DWT), and other multiresolution analysis methods. Such representations make it possible to identify transient changes associated with emotional responses that may not be evident through conventional spectral analysis [5,7].
4. **Complexity features.** These include metrics aimed at quantifying the dynamic organization of brain activity, such as spectral entropy, differential entropy, sample entropy, and other information-theoretic measures. Several studies have shown that these features can provide complementary information for discriminating emotional and cognitive states [11,44].

Feature selection depends on the available channels, signal quality, sampling rate and computational constraints, and the framework requires that this dependence be stated rather than left implicit.

#### 2.3.2. Computational Modeling

Once the feature representation has been obtained, the next step is to select models capable of estimating fear-compatible neurophysiological states. The Fear-EEG-AI Framework adopts an algorithm-agnostic view. Rather than favoring a specific technique, it proposes selecting models that simultaneously satisfy criteria of predictive performance, interpretability, computational efficiency, and generalization capacity [14].

From this perspective, modeling methods can be grouped into three broad categories:

1. **Interpretable models.** These include algorithms such as logistic regression, decision trees, and rule-based systems. Their main advantage lies in the transparency of the decisions they generate—an aspect particularly relevant in biomedical applications and decision-support systems [45].
2. **Models of intermediate complexity.** These comprise techniques such as support vector machines (SVMs), random forests, gradient boosting, and other ensemble methods. Such approaches typically offer an adequate balance between accuracy, robustness, and partial interpretability, and are widely used in EEG classification applications [6,14].
3. **Deep models.** These include architectures based on convolutional neural networks (CNNs), recurrent networks (RNN/LSTM), transformers, and hybrid models. Such approaches can learn complex representations directly from the data, although they generally require greater computational resources and additional explainability mechanisms [7,46].

The choice of model should be guided by the requirements of the application rather than by preestablished methodological preferences. In portable-monitoring scenarios, energy efficiency, latency, and local-processing capacity may be priority factors. In clinical or research applications, interpretability and explanatory capacity may become more relevant. Algorithm selection is understood as a context-dependent engineering decision, whereas the conceptual architecture remains independent of the technology employed.

#### 2.3.3. Computational Design Principles

As a result of the preceding levels, any implementation compatible with the Fear-EEG-AI Framework should seek to satisfy five computational criteria:

1. Maintain neurophysiological coherence between the biomarkers used and the brain mechanisms intended to be modeled.
2. Prioritize representations that are reproducible across subjects, devices, and experimental contexts.
3. Optimize the balance between accuracy, interpretability, and computational cost.
4. Favor architectures capable of adapting to different execution platforms, including portable systems and local-processing environments.
5. Ensure validation mechanisms that make it possible to assess the robustness, generalization, and reliability of the models developed.

The third of these criteria conceals a tension that the framework must make explicit rather than resolve by preference. Deployment-oriented design and operational viability push towards on-device inference and bounded latency, while the representation dimension of Section 3.2 rewards rigorous artifact control, whose most defensible forms are computationally the most expensive. The two requirements are not reconcilable in general, and an implementation that claims both without stating the trade-off has usually satisfied neither.

The options available differ in cost and in what each one gives up. Re-referencing and fixed spatial filtering—a Laplacian or bipolar derivation—run in constant time per sample and suppress spatially broad interference, but cannot separate a contaminating source that overlaps the band of interest. Regression against a dedicated ocular or auxiliary channel removes a specific and well-characterized artifact at negligible cost, and requires the extra channel that low-channel-count hardware often lacks. Artifact subspace reconstruction operates on a covariance statistic estimated from clean calibration data and can be applied to streaming segments, which places it in the intermediate range of cost with published evaluation against manually cleaned references [47]. Independent component analysis and multiresolution decompositions, including wavelet-based ones, offer the most complete separation and are the least compatible with embedded operation: the decomposition is estimated over a block of data, which introduces latency proportional to the block, and the component selection step is the part most often left undocumented.

The framework does not prescribe a choice among these. It requires that the choice be declared as a trade-off against a named deployment constraint—latency budget, memory, power, or available channels—and that the level of artifact control actually achieved be reported, because that is what the representation dimension scores. An implementation targeting a microcontroller and reporting lightweight spatial filtering with its limitations stated is scored on what it documents; one claiming full component-based cleaning without reporting the selection procedure or the latency it incurs is not.

From this perspective, Level 3 constitutes a computational abstraction layer designed to preserve the technological currency of the framework and to facilitate its adaptation to future generations of EEG devices, artificial intelligence algorithms, and distributed-processing platforms.

#### 2.3.4. Selection of Inference Strategies

The inference stage is the component responsible for transforming the computational representations derived from EEG signals into estimates related to fear-compatible neurophysiological states. The specialized literature has employed multiple approaches for this task, including traditional statistical models, machine learning algorithms, ensemble methods, and deep learning architectures [7,14].

The diversity of reported strategies reflects the heterogeneity of EEG-based emotion-recognition studies: differences in acquisition, preprocessing, feature representation, sample size and evaluation regime make comparisons of reported accuracies across studies weakly informative, and the progression from classical classifiers to deep architectures has not removed that obstacle [7,48]. The factors that should govern the choice are generalization capacity, interpretability, computational requirements and the deployment context, and the framework treats inference as the point at which the selected correlates, the representation and the intended use are integrated—not as an independent algorithmic decision.

The evidence synthesized in the systematic review that serves as the empirical foundation of this framework shows that algorithms such as support vector machines (SVMs), convolutional neural networks (CNNs), random forests, and hybrid models have been recurrently used to identify fear-compatible neurophysiological states from EEG signals, reporting promising results under different experimental configurations [15]. Nevertheless, the differences observed across acquisition protocols, extracted features, hardware configurations, sample sizes, and validation strategies make it difficult to establish the universal superiority of any specific technique.

For this reason, the Fear-EEG-AI Framework does not prescribe particular algorithms for the inference stage. Instead, it proposes that the selection of modeling strategies be grounded both in the available scientific evidence and in the specific characteristics of the problem to be solved. The systematic review underpinning this framework provides a synthesis of the biomarkers, algorithms, performance metrics, and experimental conditions reported in the specialized literature [15].

## 3. Principles for the Design of Deployable EEG–AI Systems

The three levels described in the previous section define the conceptual architecture of the Fear-EEG-AI Framework. For that architecture to guide the development of potentially deployable systems, however, it must be translated into design principles that operate as cross-cutting criteria during decision-making. These principles connect the neurobiological foundation, the selection of biomarkers, computational modeling, and the operational constraints that arise when an EEG–AI system is intended to function outside the laboratory.

The principles presented in this section were defined by integrating evidence from three complementary domains: (i) affective neuroscience, (ii) EEG signal processing and brain–computer interfaces, and (iii) artificial intelligence systems applied to real-world deployment settings [1,7,14]. Together, these principles do not constitute rigid rules or universal technological requirements. Their function is to provide methodological guidance for assessing whether an implementation remains coherent with the central purpose of the framework: to approximate fear-compatible neurophysiological states in an interpretable, reproducible, and operationally feasible manner.

### 3.1. Design Principles

#### 3.1.1. P1. Neurophysiological Coherence

Neurophysiological coherence establishes that every design decision must maintain a justifiable relationship with the brain mechanisms it seeks to approximate. In EEG–AI systems this principle is especially important because models can identify complex statistical patterns without those patterns having biological meaning or corresponding to the emotional phenomenon of interest. In the absence of such coherence, a classifier might learn acquisition artifacts, muscle noise, protocol biases, or between-subject differences instead of signals linked to relevant neurophysiological processes [14,45].

The framework therefore holds that predictive performance must not be the sole criterion for accepting a biomarker or a computational representation. The features used must be relatable to mechanisms described in the literature on fear, threat appraisal, emotion regulation, or cortical activation. This requirement is consistent with the indirect nature of EEG: the system does not measure emotions directly but rather electrical manifestations associated with neurocognitive processes that have been experimentally linked to affective states [1,27].

In practical terms, this principle entails justifying the selection of features—such as theta, alpha, and beta modulation, frontal asymmetry, or complexity measures—on the basis of their relationship with processes of emotional activation, vigilance, regulation, or affective–cognitive integration [29,38,39]. Neurophysiological coherence does not restrict the exploration of new biomarkers; rather, it requires that each addition be locatable within an explanatory chain connecting Level 1, Level 2, and Level 3 of the framework.

#### 3.1.2. P2. Technological Independence

Technological independence establishes that the framework should remain valid even as EEG devices, acquisition configurations, processing platforms, or artificial intelligence algorithms change. This decision responds to the heterogeneity observed in the EEG-based emotion-recognition literature, where studies vary widely in channel count, sampling rate, experimental protocols, feature-extraction strategies, and classification models [7,11,14].

The framework therefore does not presuppose specific hardware, a particular software architecture, or a mandatory algorithm. Its core components are conceptual: the neurobiological foundation, the neuroelectric correlates, the computational representation, and the deployment criteria. An implementation may use high-density clinical devices, portable low-channel-count systems, interpretable models, or deep architectures, provided that it documents its decisions and remains coherent with the proposed methodological principles.

This independence does not mean that all technologies are equivalent. Different configurations may affect signal quality, spatial resolution, temporal stability, computational cost, or generalization capacity. What the principle establishes is that such decisions belong to the implementation level and must be justified according to the context of use, without becoming structural requirements of the framework.

#### 3.1.3. P3. Deployment-Oriented Design

Deployment-oriented design requires considering, from the earliest stages, the conditions under which the system might operate outside the laboratory. Many EEG–AI systems are designed to maximize performance on databases or controlled protocols but do not explicitly incorporate constraints such as portability, latency, energy consumption, user comfort, signal stability, or local processing. This disconnect contributes to promising experimental results that do not always translate into usable solutions [11,14].

From the perspective of the Fear-EEG-AI Framework, deployment should not be added at the end of the process but incorporated as a design criterion from the selection of sensors, biomarkers, features, and models. For example, an architecture with high performance but dependent on bulky hardware, continuous external computation, or complex preparation protocols may be suitable for research yet limited for continuous monitoring or remote assistance.

This principle does not require all implementations to be immediately deployable. Rather, it proposes that the intended application scenario be declared and that technical decisions be evaluated in relation to that scenario. In this way, the framework makes it possible to distinguish between exploratory systems, experimental prototypes, pre-deployable solutions, and applications with greater operational readiness.

#### 3.1.4. P4. Inference Traceability

Inference traceability establishes that the system’s outputs must be relatable to the preceding stages of acquisition, processing, feature extraction, and modeling. In EEG–AI systems aimed at fear-compatible states, this traceability is essential because inferences may carry practical, ethical, or clinical implications. A result should not be interpreted merely as a label produced by an algorithm, but as the final link in an auditable methodological chain.

This principle becomes especially relevant in the face of complex or deep learning models, whose predictive capacity may be high but whose interpretation is not always transparent. The literature on artificial intelligence applied to sensitive domains has stressed the importance of privileging interpretable models or, when complex models are used, incorporating explanation mechanisms that allow examination of which variables contribute to the final decision [7,45].

Within the framework, traceability entails documenting how the signal was acquired, what cleaning procedures were applied, which biomarkers were selected, how the features were constructed, which model was used, and under what criteria the inference was produced. In this way, the system’s outputs cease to be solely algorithmic results and become part of a chain of evidence connecting neurophysiological observation, computational decision, and application context.

#### 3.1.5. P5. Real-World Validation

Real-world validation establishes that the evaluation of an EEG–AI system should not be limited to performance metrics obtained under controlled conditions. One of the most recurrent challenges in EEG-based emotion recognition is the gap between experimental performance and the system’s behavior when exposed to inter-subject variability, session changes, hardware differences, uncontrolled artifacts, or everyday usage conditions [11,14].

For this reason, the framework proposes understanding validation as a progressive process. An implementation may begin with tests on previously recorded databases, advance toward between-subject evaluations, continue with real-time experiments, and eventually be tested under operational conditions representative of the application scenario. Each level provides distinct evidence and must be reported explicitly.

This principle does not impose a single validation protocol. Its aim is to allow the reader to interpret the results according to the degree of realism of the evaluated conditions. Consequently, validation ceases to be understood solely as a measurement of accuracy and becomes a mechanism for characterizing the validation regime and the deployment readiness of the system.

#### 3.1.6. P6. Operational Viability

Operational viability establishes that an EEG–AI system cannot be evaluated solely by its accuracy, interpretability, or neurophysiological coherence. For an implementation to be sustainable outside the laboratory, it must also consider practical conditions such as energy autonomy, user comfort, ease of setup, acquisition stability, maintenance, connectivity, storage, local processing, and ease of use.

These constraints are especially relevant in applications involving continuous monitoring, personalized assistance, or contexts where the intervention of specialized personnel is limited. A technically accurate system that is uncomfortable, costly, difficult to configure, or dependent on complex infrastructure may have low practical utility. The framework therefore proposes that operational constraints be documented and justified from the design stage, rather than treated as secondary details addressed afterward.

Operational viability does not imply a universally optimal configuration. Each scenario may require different solutions in terms of channel count, device type, acceptable latency, energy autonomy, or processing modality. From a methodological standpoint, this principle incorporates usability feasibility as an explicit dimension of the framework and as a central criterion for estimating an implementation’s degree of readiness for real-world scenarios.

### 3.2. Operationalization, Reporting, and Evaluation of the Fear-EEG-AI Framework

Defining a conceptual–operational framework requires transforming its levels and principles into observable dimensions that can guide design, document methodological decisions, and enable the comparison of implementations developed under different technological conditions. For this reason, the reporting criteria and the operationalization of the framework are integrated into a single structure: both serve to translate general concepts—such as neurophysiological coherence, traceability, validation, or operational viability—into concrete elements that can be described and evaluated throughout the life cycle of an EEG–AI system.

Owing to the diversity of potential application scenarios, the framework does not establish universal thresholds for variables such as predictive performance, latency, energy consumption, channel count, or computational complexity. Such values depend on the context of use, the type of EEG device, the experimental protocol, the target population, and the operational requirements of each implementation. Instead of imposing fixed values, the Fear-EEG-AI Framework proposes a set of minimum documentation dimensions that make it possible to report, in a homogeneous manner, how each system was designed, validated, and prepared for possible deployment.

In the literature on EEG-based emotion recognition, brain–computer interfaces, and neuroadaptive systems, it is common to find significant differences in how biomarkers are defined, how signal acquisition and processing are implemented, how artificial intelligence models are selected, and how validation procedures are reported. This heterogeneity hampers the reproducibility of results and limits systematic comparison among proposals developed under different technological and experimental contexts.

To reduce this methodological gap, the framework incorporates an operationalization structure that translates its conceptual components into observable dimensions. This structure does not seek to impose specific EEG devices, particular algorithms, or predetermined technological configurations. Its purpose is to provide a common language for relating neurobiological foundations, neuroelectric correlates, signal representation, computational modeling, traceability, validation, deployment readiness, and ethical considerations.

Table 3 presents the dimensions proposed for operationalizing and reporting implementations compatible with the Fear-EEG-AI Framework. Each dimension is identified by the label (D1–D8) used throughout the remainder of the paper and is assigned to one of the three evaluation domains, so that the reporting structure and the interpretive grouping are read from a single table. Each dimension includes a methodological objective and a set of observable indicators that can be used both to guide system design and to document its characteristics during evaluation.

The dimensions presented in Table 3 should not be interpreted as rigid sequential stages or as mandatory technological requirements. Their main function is to provide a methodological structure that facilitates the systematic assessment of the degree of alignment between a particular implementation and the principles established by the Fear-EEG-AI Framework.

The first dimensions are closely associated with the scientific coherence of the system, ensuring that engineering decisions maintain an explicit relationship with the available neuroscientific knowledge and with the neuroelectric correlates that underpin the proposed inference. The dimensions related to neuroelectric representation, computational modeling, and traceability then characterize how EEG signals are transformed into automated inferences. Finally, the dimensions of experimental validation, deployment readiness, and ethical considerations extend the scope of the framework beyond predictive performance, incorporating elements related to robustness, generalization, operational viability, and responsible use of the technology.

Taken together, this operationalization constitutes the bridge between the conceptual architecture of the Fear-EEG-AI Framework and its practical application. It also provides the methodological basis for the evaluation and alignment mechanisms developed in subsequent sections, allowing future implementations to be described, compared, and evaluated under homogeneous criteria regardless of the specific technologies employed.

This operationalization structure seeks to contribute to the methodological standardization of future research aimed at identifying fear-compatible neurophysiological states through EEG and artificial intelligence, fostering reproducibility, comparability, and the progressive accumulation of scientific evidence.

### 3.3. Framework Evaluation Model

The operationalization presented in the previous section makes it possible to document an implementation compatible with the Fear-EEG-AI Framework through eight observable dimensions. For those dimensions to serve as a methodological analysis tool, however, they must be organized into an evaluation structure that indicates which aspect of the system is being assessed and how it relates to the framework’s principles. In this sense, the evaluation model does not constitute an independent component or an additional framework, but rather the analytical translation of the conceptual architecture, the design principles, and the operationalization dimensions previously described.

The need for an evaluative layer arises because performance metrics do not report whether the construct being predicted was defined, whether the biomarkers were justified, or whether the evidence of validity extends beyond the conditions of acquisition—a limitation that is sharper in EEG–AI systems, where the signal is indirect, variable and context-sensitive.

The eight dimensions are grouped into three evaluation domains, reported in the domain column of Table 3. Their function is to facilitate a structured reading of the system: first, whether the inference is scientifically justified; second, whether the processing and modeling are technically sound and traceable; and third, whether the validation evidence, the deployment scenario, and the ethical considerations have been sufficiently documented.

The domains in Table 3 should not be interpreted as sequential stages or as independent blocks. A methodologically sound implementation requires interaction among them. For example, a system may exhibit high predictive performance, but if it lacks neurophysiological justification or traceability, its scientific utility may be limited. Similarly, a system with clear neurobiological foundations may not be ready for real-world scenarios if its validation is restricted to highly controlled conditions or if it does not document operational and ethical constraints.

From this perspective, the evaluation model functions as the direct link between the qualitative operationalization of the framework and the quantitative mechanism of methodological alignment presented in the following section. The eight dimensions make it possible to document the implementation; the three domains make it possible to interpret its methodological profile; and the Fear-EEG-AI Alignment Index (FAI) makes it possible to summarize its degree of alignment with the framework without replacing the detailed analysis of each dimension.

#### RelationBetween Principles and Dimensions

The principles of Section 3.1 and the dimensions defined here are closely related and in four cases share a name, which invites the reading that they duplicate one another. They do not, and the distinction is the same one that governs the index: a principle is a normative commitment about the system; a dimension is a slot for what the published report allows a third party to verify. Principle P4 requires that a system’s outputs remain relatable to the stages that produced them; dimension D5 asks whether the publication documents that mapping. A system may satisfy P4 by design and still score low on D5 because the mapping was never reported, and that asymmetry is informative rather than contradictory: it locates the deficiency in the reporting rather than in the design.

Table 4 states the correspondence. It is deliberately not one-to-one. Principle P2 has no dimension, because technological independence is a property of the framework rather than of any implementation and is therefore not scored; and dimensions D3 and D4 do not descend from a single principle, since they are the technical-rigor slots that P1 and P4 constrain jointly. Making both asymmetries explicit is what closes the mapping in each direction.

### 3.4. Fear-EEG-AI Alignment Index (FAI)

The Fear-EEG-AI Alignment Index (FAI) is derived directly from the evaluation model described in the previous section. Its function is not to introduce an independent quality criterion, but to synthesize, on a common scale, the degree to which an implementation documents and satisfies the eight dimensions of the Fear-EEG-AI Framework. In this sense, the FAI should be understood as a support tool for comparing methodological profiles, identifying weak areas, and communicating the overall level of alignment with the framework.

The need for a synthetic indicator arises because traditional performance metrics—such as accuracy, sensitivity, or specificity—do not by themselves capture aspects related to methodological robustness, reproducibility, interpretability, reliability, or deployment readiness [45,49,50]. In EEG-based applications this limitation is especially relevant, since a model’s performance may depend on signal quality, biomarker selection, inter-subject variability, validation procedures, and acquisition conditions [7,12,51].

Under this rationale, the FAI operates as a composite index that aggregates the scores obtained across the eight previously operationalized dimensions. The use of composite indices makes it possible to integrate multiple variables into a reference measure to facilitate comparison, monitoring, and longitudinal analysis [52]. Nevertheless, following the methodological recommendations for this type of indicator, the aggregate value must always be interpreted together with the individual dimension scores, avoiding the reduction of a multidimensional phenomenon to a single isolated figure [52].

In its initial formulation, all dimensions receive uniform weighting. This decision responds to the absence of sufficient empirical evidence to justify differential weights among specific framework components. A conservative approach is accordingly adopted, one that avoids introducing weight differences not supported by experimental evidence [52]. Future research may explore alternative weightings through expert consensus, empirical validation, or multi-criteria analysis.

The FAI is computed through the normalized aggregation of the scores obtained across the eight dimensions considered by the evaluation model:

(1) $$\mathrm{FAI}=\left(\frac{\sum_{i=1}^{8}D_{i}}{8\times D_{\max}}\right)\times 100$$

where FAI denotes the Fear-EEG-AI Alignment Index, expressed on a normalized scale from 0 to 100 points; $D_{i}$ represents the score obtained on evaluation dimension *i*; and $D_{\max}$ is the maximum possible score for each dimension. In the present model, $D_{\max}=5$, in accordance with the proposed rating scale. The eight dimensions considered are: construct delimitation, neuroelectric correlates, neuroelectric representation, computational modeling, inference traceability, experimental validation, deployment readiness, and ethical and privacy aspects. The complete scoring rubric, with anchor descriptors for each of the eight dimensions across the five score levels, is provided in Appendix A.

Considering eight dimensions and a maximum score of five points per dimension, the maximum achievable value corresponds to forty points. Equation (1) normalizes this result to a percentage scale from 0 to 100, where higher values indicate greater methodological alignment with the Fear-EEG-AI Framework.

The FAI must be interpreted with caution. A high score indicates greater consistency with the conceptual and methodological principles of the framework, but does not necessarily imply superiority in predictive performance, clinical utility, or operational effectiveness. Likewise, a low score does not invalidate an implementation; rather, it signals dimensions where methodological documentation, experimental evidence, or deployment considerations require strengthening. This caution is consistent with the warnings about the use of composite indicators when they are employed to represent complex systems [52].

The FAI should therefore be used as a complement to the dimensional analysis and not as a substitute for it. Its main contribution lies in making the completeness of methodological documentation explicit and comparable across implementations, and in providing a common reference for research aimed at developing EEG–AI systems aligned with the principles of the Fear-EEG-AI Framework.

Two properties of this construction bound its interpretation and are stated here rather than among the limitations, because they determine what the index may be used for.

First, the FAI measures documented alignment. Each dimension is scored from the information a publication makes available, and the absence of reported information is scored as a deficiency rather than assumed in the study’s favor. A low score therefore identifies insufficient documentation or evidence; it is not a finding about the quality of the underlying system, and the index must not be read as one. The separation mirrors the one that reporting instruments such as TRIPOD+AI maintain with respect to risk-of-bias tools such as PROBAST+AI [16,17]. The index is likewise not intended, and should not be used, as an input to editorial, funding or personnel decisions.

Second, summing anchored ordinal scores assumes that the intervals between adjacent anchors are comparable across levels and across dimensions. That assumption is a property of the instrument rather than a demonstrated result, and it is why the aggregate is reported together with the dimensional profile and never in place of it, in line with established guidance on composite indicators [52]. One rubric point corresponds to 2.5 FAI points, which sets the smallest difference the index can express and bounds any comparative reading of it.

### 3.5. Sensitivity of the Index to Weighting and Rater Error

Uniform weighting is a defensible default in the absence of evidence for differential importance, but it is a specification choice, and any comparative use of the index requires evidence that conclusions do not depend on it. The behavior of the index was therefore examined under alternative weightings and under the measurement error introduced by independent raters.

Seven specifications were compared. Four are deterministic: (S1) uniform weights, as in Equation (1); (S2) equal weight to each of the three evaluation domains; (S3) a clinical–assistive scenario, weighting construct, validation, deployment and governance more heavily; and (S4) a laboratory–methodological scenario, weighting representation, modeling and traceability more heavily. Three are structural or stochastic: (S5) 10,000 weight vectors drawn from a Dirichlet(α=1) distribution over the eight-dimensional simplex; (S6) eight leave-one-dimension-out variants; and (S7) perturbation of individual rubric cells by one point, which bounds the effect a single scoring disagreement can have on the index. The weight vectors of S2–S4, fixed on substantive grounds before the analysis was run, are given as Supplementary Materials. The analysis is performed on a fixed score matrix, so it characterizes the behavior of the index specification and not the variability of the scoring process, which Section 3.9.2 addresses.

**Figure 1:**
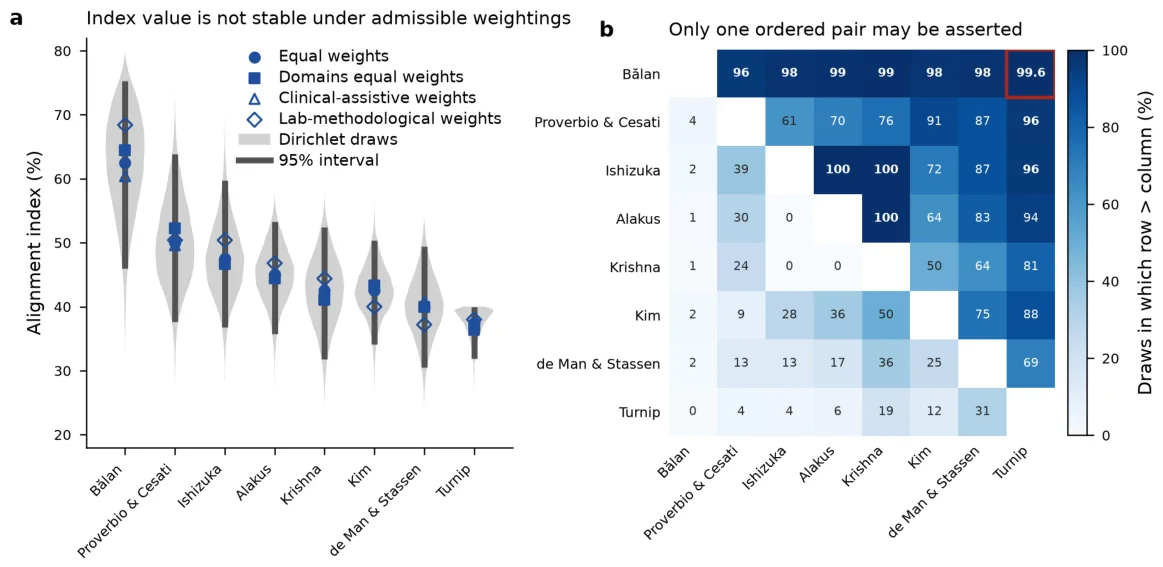
Sensitivity of the alignment index to its weighting specification, computed on the adjudicated dimensional scores of the eight eligible studies (8 dimensions)—Bălan et al. [53], Proverbio & Cesati [54], Ishizuka et al. [55], Alakus et al. [56], Krishna et al. [57], Kim et al. [58], de Man & Stassen [59], and Turnip et al. [60]—ordered by equal-weight index value. (**a**) Distribution of the index under 10,000 weight vectors drawn from a Dirichlet(α=1) distribution over the eight-dimensional simplex (shaded), with the 95% interval (vertical bar) and the four deterministic specifications S1–S4 overlaid. (**b**) Proportion of draws in which the study of each row scores above the study of each column, with proportions of 95% or above in bold; the decision rule of Section 3.5 requires this proportion to reach 95% and the corresponding intervals not to overlap. Twelve of the 56 pairs reach the proportion threshold and one satisfies both criteria.

Figure 1 reports the outcome. Under S5 the 95% intervals are wide and largely overlapping, with a mean width of 20.0 percentage points (panel a). The rank correlation with the equal-weight specification has a median of ρ=0.85 and a 95% range of 0.37 to 0.99, so a randomly chosen admissible weighting preserves the broad arrangement while rearranging its interior: the complete equal-weight ordering of the eight studies is reproduced in only 10.1% of the 10,000 draws. Orderings are compared on the 27 of 28 study pairs that the equal-weight specification orders strictly; the one pair that shares an index value is excluded, because its order is undefined and any convention for breaking it would be counted as a reversal. Of the 56 ordered pairs, 12 reach the 95% draw threshold, and only one of those—the highest-scoring study against the lowest-scoring one—also has non-overlapping intervals (panel b). Under S6, removing a single dimension shifts the index by up to 6.1 points relative to the full specification and reverses at least one strictly ordered pair in three of the eight variants. Under S7, altering one rubric cell by one point changes the index by exactly 2.5 points, which equals or exceeds the separation between six of the seven adjacent pairs in the corpus.

An explicit decision rule is adopted, fixed before the pairwise results were tabulated and stated here before they are interpreted: no difference in alignment between two implementations is asserted unless the ordering is preserved in at least 95% of the weight draws of S5 and the corresponding 95% intervals do not overlap. Where this condition is not met, dimensional profiles are reported and no ordering is claimed. Applying the rule to the present corpus, exactly one of the 56 ordered pairs may be asserted—the highest-scoring study over the lowest-scoring one, separated by 25.0 index points, equivalent to ten rubric points. Eleven further pairs satisfy the draw criterion but not the interval criterion, and the remaining 44 satisfy neither. Apparent separations of this magnitude among the highest-scoring studies are therefore not supported by the instrument that produces them.

The interval criterion is deliberately conservative, and two pairs show why. Ishizuka et al. [55] over Alakus et al. [56], and Alakus et al. [56] over Krishna et al. [57], are ordered in every one of the 10,000 draws, because in each pair one dimensional profile weakly dominates the other across all eight dimensions. Neither pair is asserted: the two profiles differ on a single dimension by a single point, which is within the disagreement the raters themselves produced—19 of the 21 adjudicated cells differed by exactly one point. A pair whose ordering rests on one rubric point is not distinguishable from one scored differently by an equally competent rater, and the criterion is set to reject it. This is why the results table below is ordered alphabetically rather than by index value, and why the index is presented throughout as a descriptor of methodological profile rather than as a ranking device.

### 3.6. Interpreting Index Values

Two questions are often conflated when a composite index is read, and the analysis of Section 3.5 answers them differently. Asking whether one implementation is better aligned than another requires resolving differences of a few index points, and the instrument demonstrably does not resolve them. Asking where an implementation sits on the scale in absolute terms is a coarser question, and it can be answered provided the resolution claimed does not exceed the resolution demonstrated.

That constraint fixes how many interpretive bands the scale admits. The mean width of the 95% interval across admissible weightings is 20.0 percentage points, so no partition of the 0–100 range into more than four bands survives a change of weighting specification. Three are defined here, because the additional boundary that a fourth would require has no basis in the present corpus. Any finer scheme would assign an implementation to different bands under specifications that are equally defensible. Three bands are therefore defined, each gated by a floor on the dimensional profile rather than by the aggregate alone:

- **Insufficiently documented**: below 40, or any dimension scored 1 irrespective of the total. The report does not support an assessment of methodological alignment, either because too little is documented overall or because one necessary element is absent.
- **Partially documented** (40 to below 70), and no dimension scored 1.
- **Substantially documented** (70 or above), and no dimension scored below 3.

The floor is what makes the band informative. Without it an implementation could reach the highest band on the strength of five well-documented dimensions while leaving a sixth at the bottom of the scale—the same pathology that the ordinal reading of the validation axis produced, and which Section 3.7 removes by construction. With it, a band is a claim about the whole profile.

The floor is the point at which the instrument stops behaving as a weighted sum. A dimension at the lowest anchor prevents a band from being assigned whatever the total, so for that dimension the criterion functions as an admissibility condition rather than as a contribution that other dimensions can offset. It is a constraint on which band may be claimed and not a finer resolution of the scale, and it does not license the ordering of two implementations, which remains governed by the criterion of Section 3.5. Applied to the corpus of Section 3.9.1, the bands do not separate the implementations. Every study records at least one dimension at the lowest anchor, so the floor assigns the whole corpus to the lowest band whatever its total, and the highest total obtained, 62.5, falls short of the threshold of the upper band in any case. The bands therefore describe a property the corpus shares rather than a gradient within it, and the demotion is informative in itself: seven of the eight studies would have qualified for the middle band on their total alone.

Three properties of these bands must be stated explicitly, because the scale invites the opposite reading. They are declared conventions and not empirical thresholds: with a corpus of this size there is no basis for calibrating cut-points against a reference distribution, and the only justification for the boundaries chosen is that the bands are wider than the measured uncertainty of the instrument. They are not performance categories: a band describes the completeness of documentation and the type of evidence reported, not the accuracy or the clinical value of the underlying system. And they do not reinstate ranking: two implementations in the same band are not thereby equivalent, and two in adjacent bands are ordered only if they also satisfy the criterion of Section 3.5, which in the present corpus a single pair does.

### 3.7. Validation Evidence Levels

The alignment index describes how completely an implementation documents its design decisions. It does not describe what kind of empirical evidence supports the inference, and the two can diverge sharply: a system may be thoroughly documented yet tested only offline, or may operate in real time while never having been evaluated across subjects.

In the field of brain–computer interfaces and EEG-based systems, several studies have noted that the performance observed under controlled laboratory conditions may differ from that obtained in operational environments, owing to factors such as inter-subject variability, the non-stationarity of EEG signals, differences between acquisition sessions, and the constraints inherent to real-world contexts of use [7,12,61].

The second axis is therefore defined as five levels of validation evidence, labeled VM1–VM5 for consistency with the systematic review from which the framework derives [15]. The levels are ordered by the strength of the evidence they denote, but they are not nested: online operation does not presuppose cross-subject evaluation, and cross-session evidence presupposes neither. Reporting a single ordinal level therefore conceals exactly the gaps the axis exists to expose—an implementation evaluated in real time but never across subjects would be assigned a high level while lacking the generalization evidence that the lower levels describe.

Five independent levels are therefore defined, each with an explicit minimum criterion, summarized in Table 5. An implementation is characterized by the full profile 〈VM1,…,VM5〉. Two summaries may be derived from it, and both must be reported together to avoid reintroducing the error just described: VM-max, the highest level attained, which is descriptive and explicitly not cumulative; and VM-core, the highest level such that all lower levels are also attained, which is the only cumulative reading the axis admits. A profile in which these two summaries differ is not an anomaly to be resolved but the principal diagnostic output of the axis.

A level is not credited when the usable sample does not support the contrast it names. Two of the levels additionally carry a composition requirement. Cross-subject generalization and operational evaluation are the two claims most often made on samples that cannot support them: a model validated across twelve undergraduates of one sex and one age decade has met the arithmetic of VM2 and not its purpose, and the affective-computing literature documents this pattern widely enough that Section 4.5 treats it as a standing risk. The levels therefore require that sample composition and recruitment base be reported for VM2, and that the deployment population and its correspondence with the target population be stated for VM5. Neither requirement prescribes a composition, because the composition that is adequate depends on the intended population; both make an inadequate one visible instead of leaving it to be inferred from a participant count. As with every other level, these are conditions on the level itself and not prerequisites for the levels above it. For VM2 the minimum was set at n≥12 participants with at least two held out, the point at which the half-width of the 95% Wilson interval for the proportion of participants above chance, estimated over the *n* participants evaluated and at a proportion of 0.8, narrows to about 21 percentage points, against 29 at n=5; the calculation is given as Supplementary Materials. Even at that threshold the estimate is coarse, which is why VM2 is binary rather than graded. Every minimum sample was fixed in the scoring protocol before the corpus was evaluated, not adjusted to the data.

The levels are stated as descriptors of evidence already obtained, which leaves open how a prototype should be moved from the weakest to the strongest of them. The framework therefore specifies what must be established before each level is credited, so that a development program can be planned against the axis rather than scored against it after the fact. Four requirements apply in the order in which evidence is normally acquired, without implying that the order is obligatory.

Before VM1. Fix the target construct and the adjacent states against which it will be contrasted, the correlates to be used and their artifact-control criterion, and the scoring of the outcome. Register them before data are seen. An offline result obtained after the analysis choices were made in view of the data credits nothing, and this is the stage at which most of the framework’s requirements are either met or irrecoverably lost.

Before VM2. Define the participant partition before training and keep it fixed, with no tuning on held-out participants, and report the sample composition required in Table 5. The comparison of interest is the performance drop between within-subject and cross-subject regimes, which should be reported rather than the cross-subject figure alone.

Before VM3 and VM4. For cross-session evidence, declare the interval between sessions and whether the montage was re-applied, since re-application is the condition under which the claim becomes informative. For real-time evidence, report end-to-end latency, decision rate and the computational configuration on which they were measured, and state whether any step was performed offline, since a pipeline with an offline artifact-rejection stage is not operating in real time.

Before VM5. Describe the deployment population and its correspondence with the intended one, record failures and not only successes, and report the governance arrangements under which the deployment took place. A system evaluated with end users in a context whose constraints are not documented has produced a demonstration rather than operational evidence.

Because the levels are independent, this sequence is a development path and not a ranking: a prototype may satisfy the real-time requirements while still failing the cross-subject ones, and the two summaries of the axis are designed to make that asymmetry visible rather than to average it away.

The profile does not replace the index: the index summarizes methodological documentation, the profile describes the type of empirical support, and their joint use distinguishes a conceptually sound but preliminary system from a technically advanced but poorly traceable one, and both from an implementation with operational evidence but limitations in neurophysiological foundation or governance.

Applied to the eight eligible studies, the profile is uniform: every study satisfies VM1 and none satisfies VM2, VM3, VM4 or VM5, so VM-max and VM-core equal VM1 throughout. The axis therefore has no discriminative power in this corpus, and the reason is not that the levels are too demanding but that the corpus is homogeneous in precisely the respect the axis measures—the evidence is retrospective and offline in every case. Two consequences follow. The first is substantive: a literature in which no implementation documents cross-subject, cross-session, real-time or operational evidence cannot support claims about deployability, however high its reported accuracies. The second is methodological: with a uniform profile the index and the profile cannot be cross-validated against each other on this corpus, and the independence of the two axes, which the construction asserts, remains to be demonstrated empirically on a corpus with variation on the validation axis.

Finally, the levels proposed here should be understood as an initial approximation derived from the literature on the progressive validation of technologies and from the challenges identified in EEG and BCI systems. Future research may refine them through empirical studies, expert consensus, or multicenter validations.

### 3.8. Integrated Architecture of the Fear-EEG-AI Framework

The integrated architecture of the Fear-EEG-AI Framework synthesizes the components developed in the previous sections within a single methodological pathway. In its final formulation, the framework is not limited to the three conceptual levels or the six design principles. It also incorporates an operationalization layer, an evaluation model, the FAI index, and the validation evidence levels. These elements serve different but complementary functions: the levels define the conceptual base, the principles guide design decisions, the dimensions operationalize reporting and evaluation, the FAI summarizes methodological alignment, and the VM levels characterize the available experimental evidence.

**Figure 2:**
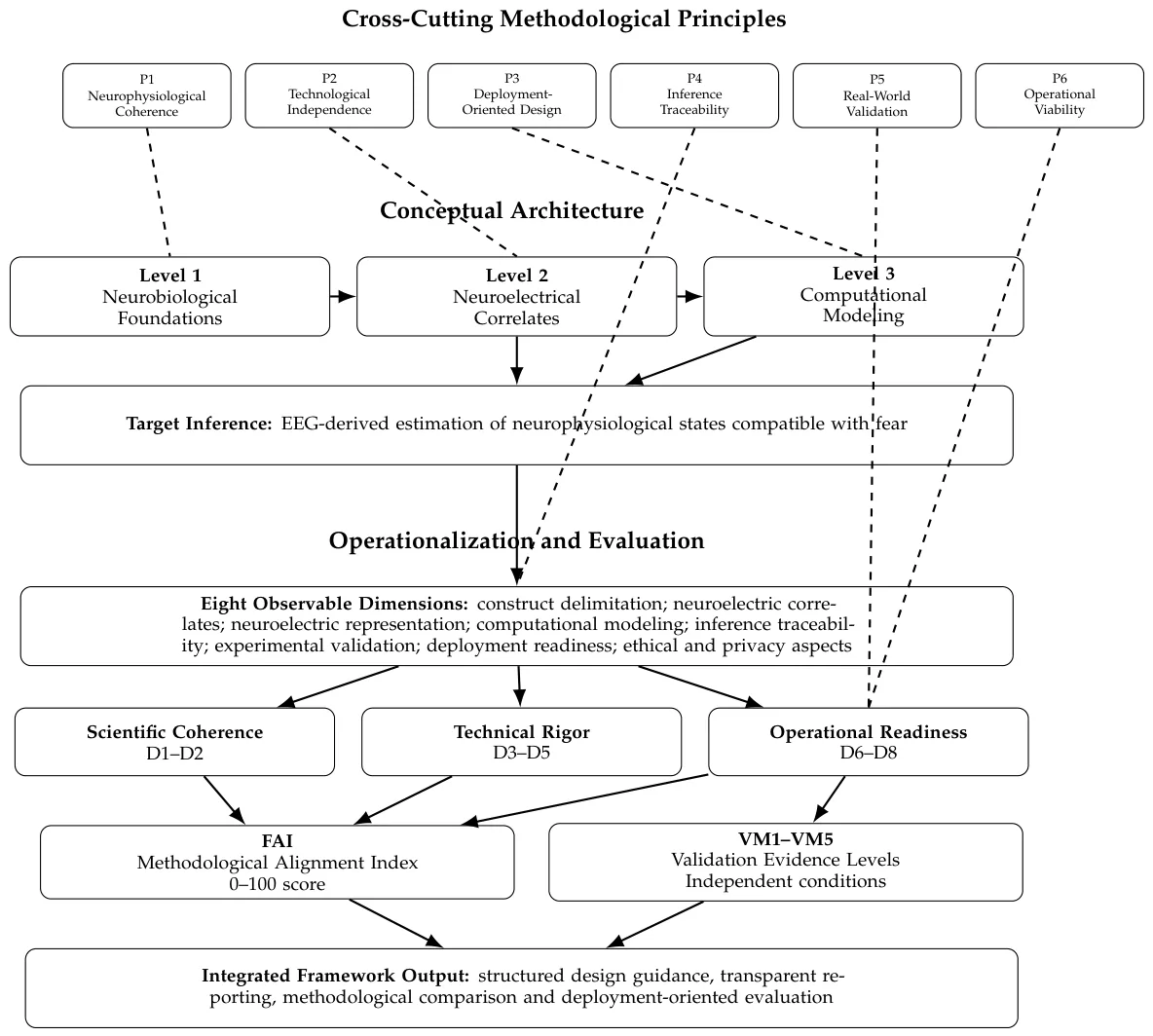
Integrated architecture of the Fear-EEG-AI Framework.The framework connects three conceptual levels, six cross-cutting methodological principles, eight observable dimensions, three evaluation domains, the Fear-EEG-AI Alignment Index (FAI), and validation evidence levels (VM1–VM5). This integrated structure supports design guidance, transparent reporting, methodological comparison, and deployment-oriented evaluation of EEG-AI systems targeting neurophysiological states compatible with fear. Solid arrows indicate the main design-to-evaluation flow linking the conceptual levels, the observable dimensions, the evaluation domains, and the two assessment instruments (FAI and VM1–VM5); dashed lines indicate which cross-cutting principle governs each component of that flow.

Figure 2 presents this integration. The upper part represents the conceptual base of the framework, composed of the neurobiological foundations, the neuroelectric correlates, and computational modeling. The six cross-cutting principles act upon this base, functioning as coherence criteria during design, validation, and eventual deployment. The lower part shows the operational translation of the framework: eight observable dimensions, three evaluation domains, the FAI as a methodological-alignment index, and the VM1–VM5 levels as a descriptor of the type of validation evidence available.

This structure prevents the framework from being interpreted as a list of isolated recommendations. On the contrary, each component occupies a specific position within the process. The conceptual levels answer what should be modeled; the principles answer how decisions should be made; the dimensions answer what should be documented; the domains answer how the implementation should be interpreted; the FAI answers how aligned the system is with the framework; and the VM levels answer what type of experimental evidence supports that alignment.

The architecture operates on two planes. The conceptual plane guides construction and justification; the operational plane makes the result documentable and comparable. A system may be strong on one and weak on the other—portable hardware with an undefined construct, or a well-grounded construct with no evidence beyond a single offline partition—and the framework is designed so that each case is visible rather than averaged away. It can therefore be used in three ways: as a design pathway for new systems, as a reporting guide for existing ones, and as an evaluation instrument for third parties.

### 3.9. Methodological Alignment Analysis: Application to the Review Corpus

The framework is applied here to a corpus of published implementations in order to characterize the behavior of its two instruments. The exercise is not a definitive empirical validation of the framework: what it establishes is how completely a defined set of existing studies documents the decisions the framework identifies as consequential, how reproducible the resulting scores are between raters, and what the alignment index and the evidence profile can and cannot support once that reproducibility is known. The corpus, the eligibility criteria and the scoring procedure are specified in Section 3.9.1.

The scores express what each publication reports, and not the scientific quality or the operational performance of the system it describes. A study may implement a sound procedure and document it incompletely, and the instrument registers the documentation in either case. This distinction governs the reading of every result in this section and is stated again, as a condition on use, in Section 4.5.

#### 3.9.1. Corpus, Eligibility Criteria and Scoring Procedure

The studies evaluated here were not selected to illustrate contrasting configurations. The corpus is the complete set of studies included in the authors’ earlier systematic review [15], which applied PRISMA 2020 with published inclusion and exclusion criteria and retained eleven studies. Eligibility for the present scoring exercise was then defined by two criteria, fixed before scoring: a study is eligible if (I1) it acquires or reuses electroencephalographic signals, since the representation dimensions are not applicable otherwise, and (I2) it is a primary implementation reporting a system, an experimental protocol or an original dataset, since a secondary study has no pipeline of its own to score.

Applying these criteria to the eleven studies of the seed corpus excluded three: two audiovisual and facial emotion databases that contain no EEG [62,63] (criterion I1) and one systematic review (criterion I2). Eight studies remained and all eight were scored; the per-study decision and its reason are reported in the Supplementary Materials. The absence of an automated inference model was deliberately not used as an exclusion criterion: a study that acquires EEG without building a classifier is eligible and scores accordingly on D4 and D5, which is the correct behavior for an index of documented completeness.

Each eligible study was scored independently by two raters with training in EEG signal processing and machine learning, neither of whom participated in drafting the rubric or its application rules, and neither of whom had access to the other’s scores or to any previous scoring of these studies. Studies were presented in a different random order to each rater. Admissible sources were the published article and its Supplementary Materials, plus the primary documentation of a reused public dataset solely to establish which processing steps were inherited rather than performed by the authors. For every cell the rater recorded the score, the anchor applied, the application rules invoked (Appendix A.2) and the supporting passage of the source. Every disagreement was resolved by a third rater against the literal anchor descriptors and the mandatory application rules, recording the anchor applied and the rule invoked in each case; agreement statistics are computed on the scores prior to adjudication. The rubric, its application rules, the scoring protocol and the eligibility decisions were finalized before scoring began and are reproduced in the Supplementary Materials in the form in which they were applied, together with the rule invoked and the basis of each adjudicated decision, so that the instrument as applied can be compared against the scores it produced. This sequence is documented but not independently timestamped: it rests on the authors’ account and on the internal consistency of the materials, not on a third-party record, and Section 4.5 states that limit.

#### 3.9.2. Inter-Rater Agreement and Composite Index Computation

Agreement was quantified over the pooled set of 64 study-by-dimension pairs with Cohen’s κ under three weighting schemes—unweighted, linear and quadratic, the last appropriate for ordinal scores in that it penalizes larger disagreements more heavily—and for the complete dimensional profile with the two-way intraclass correlation coefficient, reported both for a single rater, ICC(2,1), and for the mean of two, ICC(2,*k*). Per-dimension κ was not estimated: with eight studies each dimension contributes only eight paired observations, too few for a stable coefficient, so per-dimension agreement is reported as exact agreement and mean absolute deviation instead. The composite index was then computed with the uniform-aggregation model of Equation (1), and its behavior under alternative weightings is reported in Section 3.5.

Across the 64 pairs the two raters agreed exactly in 43 cases (67.2%) and to within one point in 62 (96.9%), with a mean absolute deviation of 0.359 rubric points. Nineteen disagreements were of one point and two of two points; none exceeded two. Pooled agreement was κ= 0.539 unweighted, 0.636 under linear weighting and 0.741 under quadratic weighting, and the dimensional profile gave ICC(2,1) = 0.744—the coefficient that applies to the scores used here, which are adjudicated values rather than rater means—and ICC(2,*k*) = 0.853. Twenty-one cells required adjudication, all resolved by rereading the descriptor and applying the rules of Appendix A.2; no disagreement was settled by averaging the two ordinal scores. Table 6 reports agreement by dimension. On the validation axis, where the decision is binary, the raters agreed on 36 of 40 level assignments (90.0%, κ=0.615); all four disagreements fell on VM1 and all were resolved in favor of crediting the level, since VM1 requires a reported offline evaluation regime with metrics and not necessarily a machine-learning classifier. VM2 to VM5 were assigned identically by both raters in all eight studies.

The three dimensions with the lowest exact agreement are construct delimitation, inference traceability and experimental validation, each at 50%, and the disagreements are not randomly distributed. One recurring source accounts for six of the 21 disagreements: one rater assigned the lowest anchor to studies reporting no classifier in inference traceability and experimental validation, whereas the instrument admits the second anchor whenever a documented processing pipeline or a formal offline evaluation is present; in a smaller number of cells the same rater assigned the upper anchor where an explicit condition demanded by that anchor was absent from the source. Both patterns are readings of one ambiguity—whether an anchor describes what a study achieved or what it documented—which the application rules of Appendix A.2 resolve in favor of what is documented.

One feature of the adjudication should be stated explicitly, since it is visible in the record. In twenty of the 21 adjudicated cells the resolved value coincides with the score of the second rater, and in the remaining cell—the one rule R2 governs—with that of the first. The adjudication record attributes this to those scores fitting the literal descriptors and the mandatory rules, which is consistent with the disagreements being concentrated in the two readings the rules exclude, both of them the first rater’s. Two qualifications follow and are stated rather than left to inference. The adjudicator was not blind to which rater had produced which matrix, so the concentration cannot be shown to be independent of that knowledge. And the adjudicated matrix should accordingly be read as the output of the rules applied to two independent readings, not as a consensus between them. The agreement statistics are unaffected, being computed before adjudication.

The residual disagreement is dimension-specific. In construct delimitation it turns on whether naming the target state and citing threat circuitry amounts to delimiting that state against adjacent ones such as stress, anxiety or generalized arousal; the adjudication required explicit delimitation for the third anchor. In inference traceability rule R4 was decisive, since describing a processing pipeline, or appealing to the structural interpretability of a model family, does not demonstrate which features produce a decision; this dimension carries the highest mean deviation of the eight. In experimental validation the question is whether a formal offline evaluation that neither employs a classifier nor breaks subject dependence should be read as anecdotal or as documented but limited, which the anchors of $D_{6}$ separate on the presence of reported metrics. The two dimensions with the highest agreement, deployment readiness and ethical provisions, are those whose anchors ask whether a specific item is present in the text, which is the form of anchor that transfers between raters with least interpretation.

Two limitations of this procedure are declared. The raters are co-authors of the manuscript proposing the instrument; agreement is therefore internal, and external replication by raters unconnected to the framework remains necessary. And with eight studies the agreement estimates carry wide uncertainty, which is reported rather than smoothed.

Two caveats bound the interpretation of the resulting scores. First, the rubric scores the quality and completeness of what each study reports; a low score signals insufficient documentation or evidence, not necessarily an inferior underlying system. Second, publication format is a confound, since short-format sources have less room to document decisions; the format of each source is recorded. Table 7 reports the adjudicated dimensional scores, the index values and the evidence profiles of the eight eligible studies. This matrix allows methodological profiles to be compared but does not establish a ranking of the state of the art, for the reasons given in Section 3.5.

The matrix below replaces the four hand-selected case studies of the previous version with the eight eligible studies of the corpus, scored under the protocol and the application rules by two raters and adjudicated by a third. The scores differ from those of the previous version; the four studies common to both are re-scored under the rules.

#### 3.9.3. Dimensional Reading of the Corpus

Table 7 carries the dimensional profile of each implementation; read by dimension rather than by study, the eight profiles show a pattern that the aggregate conceals.

No dimension reaches a mean of 3 across the corpus. The lowest is ethical and privacy provisions ($D_{8}$, mean 1.50), on which no study exceeds 2: baseline compliance or no report at all predominates, and privacy-by-design and the explicit management of false positives and negatives are practically absent. The second lowest is experimental validation ($D_{6}$), whose behavior is the most informative result of the exercise: all eight studies score exactly 2. The dimension has zero variance and therefore contributes nothing to any difference between implementations in this corpus. Every study documents a formal offline evaluation, and none documents a regime that breaks subject dependence, session dependence or offline operation. The highest mean, inference traceability ($D_{5}$, mean 2.75), still falls below the midpoint of the scale.

Every one of the eight studies scores 1 on at least one dimension. Under the admissibility floor of Section 3.6 this places the entire corpus in the lowest interpretive band irrespective of its index value, so the band scheme has no discriminative power here. That is a finding about the corpus rather than a defect of the scheme, and it is the expected behavior of an instrument that scores documentation rather than performance, applied to a literature that reports classifier results more completely than construct definitions, validation regimes or governance provisions.

Read by study, the profiles are asymmetric rather than ordered. The highest-scoring implementation is the only one reaching anchor 4 on five dimensions, and it does so partly on inherited preprocessing, capped by rule R3; it nonetheless scores 1 on ethical provisions. The lowest-scoring implementation presents a different problem, which the rubric registers as low traceability and construct delimitation but which the raters identified explicitly: its fear classes are constructed from the same EEG amplitudes that subsequently feed the classifier, so the target is circular and the reported accuracy cannot be read as evidence of discrimination. A reporting instrument can require the documentation that makes such a construction visible; it cannot by itself detect the circularity, and that limit is stated in Section 4.5.

Two further observations recur across the corpus and are captured by no single dimension. First, segmenting one recording into many analysis windows inflates the number of computational samples without increasing the number of biologically independent observations, so reported sample sizes are not comparable between studies without knowing the unit of replication. Second, ocular and muscular artifact control is insufficient in several sources, which matters most where the inference rests on frontal electrodes or on components above 30 Hz—the condition that rule R2 caps and that Section 2.2.6 makes auditable.

The adjudicated value of all 64 study-by-dimension cells, and for each of the 21 cells in which the raters differed the score of each rater, the rule invoked and the basis of the decision, are provided as Supplementary Materials; in the 43 remaining cells both raters recorded the value shown in the matrix, so the two individual sets of scores can be reconstructed in full from what is supplied.

#### 3.9.4. Global Discussion and Synthesis

The cross-analysis of the eight eligible studies shows how the Fear-EEG-AI Framework, together with the alignment index and the evidence profile, organizes a multidimensional reading of heterogeneous implementations. Rather than reducing the comparison to predictive performance (accuracy), the exercise makes it possible to observe engineering tensions and trade-offs that tend to remain hidden when systems are compared solely through classification metrics.

The dimensional reading of Section 3.9.3 gives the substance of that illustration: a validation dimension on which all eight studies score identically and which therefore cannot distinguish them, and a governance dimension on which no implementation exceeds 2. Both are properties of what the set reports, not verdicts on the systems, and both are the kind of finding that a performance metric cannot express.

In summary, the application of the index and the evidence profile to the corpus shows that the framework can function as a retrospective analysis instrument and as a methodological guide for identifying strengths and areas for improvement in EEG–AI systems. It does not constitute a definitive validation of the framework: it characterizes the instruments on one small corpus, and its main value lies in showing how the proposed architecture organizes the discussion of neurobiological foundation, algorithmic traceability, validation, and operational viability—and in establishing, quantitatively, the limits within which the resulting scores may be read.

## 4. Discussion

The reviewed evidence confirms that the identification of fear-compatible neurophysiological states through EEG and artificial intelligence has reached a considerable technical level: there are plausible biomarkers, consolidated feature-extraction strategies, and models capable of achieving competitive performance on databases or controlled experimental protocols [11,15,46]. However, this same evidence reveals a structural weakness that cannot be resolved solely by increasing classifier accuracy: much of the literature continues to evaluate systems under conditions that favor experimental performance but do not adequately represent the neurophysiological, operational, ethical, and social constraints of real-world deployment.

This distinction is central to interpreting the contribution of the present work. An EEG–AI system may achieve high accuracy under cross-validation and still lack sufficient neurobiological grounding, depend on poorly portable hardware, use non-generalizable induction protocols, operate as a black box, fail in the face of everyday artifacts, or lack safeguards for sensitive neural data. The open problem is therefore not solely algorithmic; it is a problem of methodological alignment among neurobiological construct, electrophysiological representation, computational inference, validation, and deployment.

The Fear-EEG-AI Framework positions itself precisely in that gap. Its contribution does not consist in proposing a new biomarker, a deep learning architecture, or a closed electrode configuration, but in offering a structure for subjecting each technical decision to a more demanding question: does this decision contribute to building a system that is neurophysiologically interpretable, technically reproducible, operationally viable, and ethically defensible? This question shifts the criterion of success from the isolated optimization of a metric toward a multidimensional evaluation of the system as a scientific and technological artifact.

### 4.1. From Experimental Accuracy to Deployment Evidence

The literature on brain–computer interfaces and machine learning applied to EEG has recurrently shown that the performance obtained in the laboratory does not guarantee robustness under real-world usage conditions [7,14,51,64]. Inter-subject variability, signal non-stationarity, artifact contamination, temporal drift, user fatigue, device adaptation, and context dependence modify the data distribution on which classifiers operate. Consequently, a validation based solely on internal partitions of a single database may overestimate the system’s ability to generalize to new users, new environments, or prolonged periods of operation.

This point refutes a frequent interpretation in the EEG-based emotion-recognition literature: assuming that high accuracy on standardized databases equates to evidence of applicability. Public databases and controlled protocols are indispensable for comparing methods, but they do not replace prospective, external, longitudinal, and context-oriented validation. From this perspective, the framework proposes that the relevant question be not only “which model classifies best?”, but “under what conditions does that inference remain valid, explainable, and actionable?”.

The implication is especially important for fear-oriented systems. Fear is not directly observed in the EEG; what is recorded are cortical dynamics that may be compatible with processes of vigilance, threat appraisal, attention, autonomic arousal, or emotion regulation. Therefore, claiming that a system “detects fear” without delimiting the neurobiological construct leads to overinterpretation. Level 1 of the framework corrects this risk by requiring a prudent definition of the inferential target: to approximate fear-compatible neurophysiological states, not to diagnose a subjective experience in absolute terms.

### 4.2. Methodological Contribution Relative to Algorithm-Centered Approaches

A strong contribution of the framework is that it reorganizes the field around a chain of validity. Level 1 defines the neurobiological construct; Level 2 translates that construct into observable electrophysiological correlates; Level 3 transforms those correlates into computational representations and automated inferences. This architecture prevents the model from learning spurious associations disconnected from the phenomenon of interest—a widely recognized risk in high-dimensional machine learning systems [7,12].

The proposal also introduces a necessary critique of the excessive reliance on complex models. Deep learning approaches have expanded classification capabilities in EEG, but their use must not be confused with automatic methodological superiority [7,46]. In sensitive applications, a less complex but interpretable, reproducible, and on-device-executable model may be scientifically preferable to a black-box architecture with marginally higher accuracy. This position is consistent with recent arguments in high-stakes artificial intelligence, where the indiscriminate use of opaque models has been questioned when sufficiently competitive interpretable alternatives exist [45].

The framework does not reject complex models; it conditions them. A deep architecture may be appropriate if it demonstrates traceability, external robustness, bias control, computational efficiency, and data governance. In the absence of these conditions, algorithmic complexity becomes technical and scientific debt: it increases maintenance costs, hampers auditing, limits reproducibility, and may conceal spurious dependencies on the experimental protocol [49,50]. This is a critical discussion for a field in which accuracy increases are often presented as advances, even when they do not improve deployment capability.

### 4.3. What the Application of the FAI and the Validation Evidence Levels Reveals

The retrospective application of the Fear-EEG-AI Alignment Index (FAI) and the validation evidence profile should not be interpreted as a definitive validation of the framework, but as a demonstration of its analytical usefulness. Its value lies in making visible tensions that traditional metrics tend to conceal. A study may be sound in neuroelectric representation and internal validation yet weak in traceability, neurobiological foundation, or deployment readiness. Likewise, a portable implementation may appear promising from an engineering standpoint yet remain insufficient if it lacks sample size, external validation, or ethical governance.

This reading makes it possible to refute another frequent premise: that EEG–AI systems can be adequately compared through a single performance metric. Multidimensional comparison reveals that the quality of a system does not depend solely on its accuracy, but on the coherence among its objectives, signals, models, validation, and usage conditions. In this sense, the FAI functions as a matrix for methodological discussion rather than as a definitive quality index. Its initial purpose is to organize the evidence, identify gaps, and guide the design of future studies.

Nevertheless, this contribution must be kept within prudent limits. The FAI adopts uniform weighting of its dimensions, which favors transparency and simplicity but does not imply that all dimensions carry equal importance in every scenario. In clinical or assistive applications, for example, external validation, safety, traceability, and error management may carry greater weight than the marginal optimization of accuracy. Future versions should therefore explore weightings based on expert consensus, multi-criteria analysis, and accumulated empirical evidence, following good practices for composite indicators and technology-maturity models [18,19,52].

### 4.4. Implications for Design, Validation, and Evaluation

The framework implies that the design of EEG–AI systems should begin with deployment criteria from the earliest stages, rather than incorporating them at the end as secondary constraints. The selection of channels, frequency bands, time windows, preprocessing methods, classification models, and computing architecture must be evaluated as an interdependent system. A decision that improves laboratory accuracy may worsen portability, increase latency, reduce interpretability, or heighten the risk of data leakage.

In terms of validation, the framework requires moving from a logic centered on internal performance toward a logic of progressive evidence. Cross-validation remains useful but must be complemented by cross-subject testing, external validation, temporal evaluation, artifact analysis, uncertainty estimation, and tests under representative conditions. In systems intended to operate with vulnerable people, the confusion matrix is not merely a statistical result: a false negative may delay a necessary intervention, whereas a false positive may generate anxiety, alarm fatigue, loss of autonomy, or unwarranted interventions.

This discussion also affects the selection of metrics. Accuracy, sensitivity, specificity, AUC, and F1-score are necessary but insufficient. They must be accompanied by metrics of latency, energy consumption, temporal stability, probabilistic calibration, explainability, low-confidence rejection rate, and subgroup performance. The evaluation of a fear-oriented EEG–AI system must answer not only whether the model predicts, but when it fails, in whom it fails, why it fails, and what consequences the error produces.

### 4.5. Technical Limitations and Refutability Criteria

The main technical limitation of the framework is emotional specificity. Although the neurobiological anchoring offers a more prudent alternative than purely dimensional affective models, EEG does not allow fear, acute stress, intense anger, surprise, cognitive load, or nonspecific physiological arousal to be distinguished unequivocally. The frontal, frontotemporal, and oscillatory correlates described in the literature are conditions of plausibility, not direct proof of subjective experience. Any implementation must therefore explicitly report the space of emotional confusion considered and avoid claims of absolute detection.

A second limitation derives from the trade-off between portability and signal quality. The use of low-cost devices or reduced configurations may favor deployment, user acceptance, and edge processing, but it increases exposure to ocular, muscular, movement, poor-impedance, and environmental-noise artifacts. This point is critical because some features associated with emotional activation may be confounded with muscle activity or peripheral contamination, especially in frontal regions [40]. Operational viability must therefore be assessed together with strict controls for preprocessing, artifact detection, and signal-quality estimation.

A third limitation concerns demographic generalization. Many EEG studies and affective-computing databases rely on small, young, university-based, or culturally homogeneous samples. A system trained under these conditions may fail in older adults, clinical populations, people with neurological conditions, medicated users, culturally diverse groups, or communities with low technological familiarity. This threat is not minor: in applied AI, data biases can translate into systematic errors and unequal consequences across groups [65]. Consequently, subgroup validation should not be optional when the system aspires to deployment.

A fourth limitation concerns the instrument rather than the framework. Because the rubric scores what a publication reports, publication format is a confound: short-format sources have less room to document decisions and are penalized by construction. The direction of this effect is predictable, but its magnitude is not estimable from a corpus of this size, and scores are therefore not adjusted for format. This property is shared by all reporting-completeness instruments, and it is why the index must be read together with the dimensional profile and never as an appraisal of the underlying systems.

A further limitation concerns modality. The framework is unimodal by design: it governs inference from neuroelectric activity and says nothing about the fusion of EEG with peripheral autonomic, thermal or behavioral channels, which the literature cited in Section 1 shows to be an active and effective alternative. Two consequences follow. The dimensions concerning correlates and representation (D2, D3) have no counterpart for non-neural channels and would have to be extended, not reused, before the framework could be applied to a multimodal system; and the specificity problem that motivates D2 is not solved by adding modalities, since peripheral and thermal signals are themselves shared across negatively valenced states of high arousal. Whether a multimodal extension should add dimensions or add a second profile per modality is left open here.

A fifth limitation concerns the corpus. The seed corpus derives from a search strategy anchored on the term fear detection, which plausibly omits relevant work indexed under threat processing, anticipatory anxiety or fear conditioning. The eight eligible studies are therefore not a representative sample of EEG-based threat research, and the descriptive patterns reported here are bounded by that corpus. A broader search is required before any claim about the state of the field as a whole can be sustained.

A sixth limitation is the most consequential, and the application of the instrument to the corpus made it visible. The rubric scores whether a study documents the provenance of its target labels; it does not and cannot establish that those labels are independent of the signal used to predict them. One implementation in the corpus constructs its fear classes from the same EEG amplitudes that subsequently feed its classifier, so its reported accuracy measures the recovery of a thresholding rule rather than the discrimination of a neurophysiological state. The rubric registers that study as weakly documented on construct delimitation and traceability, which is the correct reading of what it reports, but a reader who consulted only the index would not learn that the target is circular. Detecting target circularity requires reading the label-construction procedure against the feature-extraction procedure, which is an act of appraisal and not of reporting assessment. It is recorded here as a condition on use: the instrument is a screen that identifies where documentation is missing, and it does not substitute for methodological appraisal of the studies it scores.

A seventh limitation concerns the evidence for the chronology itself. The instrument, its application rules and the scoring protocol were fixed before the corpus was scored, and all of them are supplied as Supplementary Materials in the form in which they were applied, so a reader can check that the rules invoked in the adjudication record are the rules as written. What a reader cannot check independently is the order of those two events, because the materials were not deposited in a public repository with a third-party timestamp. The claim that the anchors were not adjusted to the scores therefore rests on the authors’ account and on the internal consistency of the record, and not on external verification. This is stated plainly because the objection it answers—that an instrument validated by its own authors on its own corpus risks circularity—is not fully answered by it. Independent application of the instrument by raters unconnected to the framework, on a corpus they select, remains the test that would settle the question.

These limitations do not weaken the framework; they delimit its conditions of refutability. An implementation aligned with Fear-EEG-AI should be called into question if it does not improve traceability, does not increase external robustness, does not reduce operational risks, or does not enable design decisions more justifiable than those of conventional approaches. The scientific strength of the proposal will depend precisely on subjecting it to prospective comparisons, multicenter studies, and longitudinal evaluations capable of confirming, adjusting, or refuting its principles.

### 4.6. Ethical, Social, and Governance Considerations

The deployment of EEG–AI systems aimed at fear-compatible states introduces challenges that exceed classification engineering. EEG signals can support inferences about cognitive, emotional, and behavioral states; their use therefore requires specific safeguards related to mental privacy, autonomy, consent, equity, and accountability [66,67,68,69]. In this domain, ethics is not a normative add-on that follows technical development; it is a design condition.

On-device processing should be regarded, whenever technically feasible, as a protection strategy and not merely as a computational optimization. Reducing the transmission of raw signals, minimizing data, controlling access, logging uses, encrypting storage, and limiting purposes are fundamental requirements for preventing surveillance, re-identification, secondary extraction of information, or unauthorized use of neurophysiological data. This requirement is even greater in continuous monitoring, where longitudinal accumulation may reveal sensitive patterns of health, behavior, or vulnerability.

User autonomy must also be preserved. An alert system for caregivers, institutions, or emergency services may be useful, but it may also become paternalistic if it turns the person into a passive object of surveillance. When no immediate risk exists, implementations should incorporate mechanisms for confirmation, cancellation, human review, and explanation of the alert. In this way, the system should operate as support for human agency, not as an automatic substitute for the judgment of the user or the professional.

Equity constitutes another central criterion. A portable, low-cost technology may democratize access, but it may also widen inequalities if it requires permanent connectivity, frequent sensor replacement, digital literacy, or specialized support. Moreover, if the models are not validated in diverse populations, they may produce differential performance and unfair decisions. Any responsible deployment should therefore report sample composition, subgroup performance, usage limits, update procedures, and accountability mechanisms.

Finally, prohibited or high-risk uses must be delimited. Technologies capable of inferring affective states should not be transferred without restrictions to labor, educational, insurance, judicial, commercial, military, or political contexts, where they could be used for surveillance, manipulation, discrimination, or coercive assessment. The framework must therefore be understood as an architecture for justified, consented, and auditable applications, not as a general license to infer emotions from the brain.

These limits are no longer solely ethical recommendations. Regulation (EU) 2024/1689 prohibits the placing on the market and the use of emotion-recognition systems in the areas of workplace and education, with narrowly drawn exceptions for medical and safety purposes, and classifies further affective-inference applications as high risk, with corresponding obligations of risk management, data governance, technical documentation and human oversight [25]. An EEG–AI system targeting fear-compatible states falls within the scope of that regime in most of the deployment contexts discussed here. Declaring the intended deployment scenario, which the framework requires on design grounds, is therefore also what determines the regulatory obligations attaching to an implementation, and should be reported as such.

### 4.7. Future Research Directions

The next scientific stage must be prospective. It is necessary to design studies that compare systems developed under the framework’s principles against systems built through conventional approaches, evaluating not only accuracy but also traceability, external robustness, efficiency, acceptability, safety, bias, and operational viability. Without this comparison, the framework will remain a plausible methodological proposal rather than empirical evidence of superiority.

Longitudinal studies in semi-naturalistic or real-world environments are also required. The stability of biomarkers, the need for recalibration, sensor degradation, habituation to stimuli, circadian variation, everyday stress, and user–device interaction can only be adequately assessed over extended periods. Such studies will make it possible to determine whether the five levels capture the distinctions that matter for deployment or whether they should be adjusted with criteria more specific to clinical, assistive, or continuous-monitoring contexts.

Finally, the framework should be evaluated on multimodal architectures and in diverse populations. Integrating EEG with autonomic signals, behavior, environmental context, or self-report may improve emotional specificity, provided that it does not disproportionately increase privacy risks or operational complexity. Likewise, validation in older adults, clinical populations, culturally diverse groups, and users with different levels of technological access will be indispensable for determining whether the proposal genuinely contributes to fairer, more robust, and more deployable EEG–AI systems.

## 5. Conclusions

Research on the identification of fear-compatible neurophysiological states through EEG and artificial intelligence has advanced considerably, yet it remains fragmented. The literature offers numerous biomarkers, signal-processing strategies, and modeling approaches, but this diversity coexists with pronounced methodological heterogeneity and limited attention to the conditions under which such systems could operate beyond controlled laboratory settings. The central problem is not, then, a shortage of algorithms, devices, or biomarkers, but the absence of a shared design rationale that connects neurobiological validity, computational traceability, and deployment feasibility.

The Fear-EEG-AI Framework was proposed to address this gap. Rather than prescribing a specific device, biomarker, or algorithm, it organizes design decisions along three interdependent levels—neurobiological foundations, neuroelectric correlates, and computational modeling—governed by six cross-cutting principles: neurophysiological coherence, technological independence, deployment-oriented design, inference traceability, real-world validation, and operational viability. This structure reframes the criterion of success, shifting it from the isolated optimization of predictive accuracy toward a multidimensional evaluation of the system as a scientific and technological artifact.

Beyond that architecture, the framework contributes operational instruments: an eight-dimension reporting scheme, a composite alignment index whose resolution is characterized rather than assumed, and five levels of validation evidence reported as an independent profile rather than as a ladder.

These contributions are conceptual and carry explicit limits. The framework does not resolve emotional specificity: no electrophysiological signature is known that isolates threat appraisal from generalized arousal, and the instruments therefore score what a report allows a third party to verify, not the quality of the underlying system. The index does not support the ordering of individual implementations, the corpus on which it was exercised is small and drawn from a narrow search, and the raters are not independent of the authors. Each of these is stated where it applies.

Understood in these terms, the Fear-EEG-AI Framework is best read as a starting point rather than a finished solution: a methodological foundation for building EEG–AI systems that are more interpretable, reproducible, ethically defensible, and prepared for the transition from the laboratory toward real, affordable, and human-centered applications.

## Figures and Tables

**Table 1:** Related reporting and readiness frameworks and their relationship to the Fear-EEG-AI Framework.

Framework	What it Covers	Relation to Fear-EEG-AI
TRIPOD+AI; PROBAST+AI	Development, validation, transparency and risk-of-bias appraisal of predictive models, independent of modality	Adopted for D4 and D6; construct-agnostic, so neither assesses whether the predicted state is identifiable
CONSORT-AI; SPIRIT-AI; DECIDE-AI	Trial design, protocols and early-stage clinical evaluation of AI interventions	Applies to a stage beyond current EEG–AI fear research; informs the upper validation-evidence levels
COBIDAS MEEG; EEG-BIDS	Acquisition, montage, referencing, preprocessing, artifact handling and data organization for EEG/MEG	Adopted, not replaced: D3 defers to these for technical detail
TRL; SRL	Progression of an artifact from concept to operational deployment	Progression logic adopted for the validation-evidence levels; artifact readiness is not evidence for an inferential claim
Regulation (EU) 2024/1689 [25]	Prohibited and high-risk uses of emotion recognition	Delimits admissible deployment scenarios; incorporated into D8
Fear-EEG-AI (this work)	Delimitation of the target affective construct, construct-to-correlate justification, and the deployment/signal-quality trade-off in portable EEG	Domain specialization inheriting from the above and addressing the three gaps they leave open

**Table 2:** Delimitation of the fear-compatible state from adjacent affective and cognitive states.

State	Typical Induction	Reported EEG Correlates	Overlap	Discriminating Evidence
Imminent threat (target construct)	Aversive images or film, virtual height, startle probes	Alpha suppression; beta increase; frontal midline theta; frontal asymmetry shifts	—	—
Anticipatory anxiety	Threat-of-shock, uncertain-cue paradigms	Frontal midline theta increase; alpha suppression	High	Temporal separation of anticipation from stimulus onset; cue predictability reported
Acute stress	Social-evaluative or arithmetic stressors	Alpha suppression; beta increase; frontal asymmetry	High	Concurrent autonomic measures; stimulus-locked rather than tonic time course
Sustained vigilance	Monitoring and detection tasks	Alpha suppression; beta increase	High	Task-load manipulation orthogonal to threat value
Cognitive load	*n*-back, arithmetic, dual task	Frontal midline theta; alpha suppression	High	Load manipulated at constant affective content
Negative valence at high arousal, including anger	Provocation, insult or aversive non-threat stimuli	Frontal asymmetry, with relative left-frontal activation under anger; beta increase	High	Valence, arousal and approach direction reported separately from threat appraisal
Panic and interoceptive threat	Interoceptive challenge; spontaneous episodes in clinical samples	The same non-specific markers: alpha suppression, beta increase, frontal asymmetry	High	Source of the threat cue reported as interoceptive rather than exteroceptive; concurrent autonomic measures; clinical status of the sample
Startle/orienting	Abrupt intense stimuli	Transient broadband and high-frequency increase	Partial	Latency characterization; transient separated from sustained state
Muscular and ocular artifact	Facial tension, frowning, blinking	Broadband high-frequency increase, frontally maximal	High	Documented EMG/EOG control; rejection or decomposition evidence

**Table 3:** Evaluation domains, observable dimensions, objectives and indicators of the Fear-EEG-AI Framework.

Domain	Dimension	Objective	Observable Indicators
Scientific coherence	**D1** Construct delimitation	Define the target neurophysiological construct and ensure coherence with evidence from affective neuroscience.	Explicit definition of the fear-compatible state; delimitation from anxiety, stress and other negative emotions; declared emotional-confusion space; identification of brain circuits related to threat processing; neuroscientific justification of the inference.
Scientific coherence	**D2** Neuroelectric correlates	Translate neurobiological mechanisms into observable, physiologically interpretable EEG biomarkers.	Alpha suppression; beta activity; frontal theta activity; frontal asymmetry; complexity metrics; inter-band ratios; physiological evidence supporting biomarker selection; stated specificity limits and artifact susceptibility.
Technical rigor	**D3** Neuroelectric representation	Ensure that EEG signals are acquired, processed, and represented consistently with the system’s objectives.	Channel count; electrode placement; sampling rate; signal quality; experimental protocol; artifact control; filtering; segmentation; normalization; extraction of spectral, temporal, time–frequency, and complexity features.
Technical rigor	**D4** Computational modeling	Generate automated inferences while balancing predictive performance, interpretability, and computational feasibility.	Algorithm selection; training strategy; validation; performance metrics; explainability; robustness; cross-subject and cross-session generalization; computational cost.
Technical rigor	**D5** Inference traceability	Relate each generated inference to the biomarkers and features that produced it.	Signal–feature–model–result mapping; feature-importance analysis; explainability mechanisms; documentation of the decision flow.
Operational readiness	**D6** Experimental validation	Evaluate the system’s ability to identify fear-compatible states under controlled and progressively more realistic conditions.	Offline validation; cross-subject validation; cross-session validation; real-time validation; classification metrics; error analysis; temporal stability.
Operational readiness	**D7** Deployment readiness	Determine the feasibility of transferring the system from the laboratory to real application contexts.	Latency; energy consumption; connectivity; robustness to noise; ease of use; maintenance; scalability; continuous-operation capacity.
Operational readiness	**D8** Ethical and privacy aspects	Ensure the responsible use of neurophysiological inferences and the protection of users.	Informed consent; EEG-data protection; transparency of inferences; management of false positives and false negatives; delimitation of clinical scope; protection of vulnerable populations; admissibility under applicable regulation.

**Table 4:** Correspondence between the cross-cutting principles and the observable dimensions. Principles state requirements on the system; dimensions state what the report is examined for.

Principle	Dimension	What the Principle Requires of the System	What the Dimension Asks of the Report
P1. Neurophysiological coherence	D1, D2	Every design decision keeps a justifiable relationship with evidence on threat processing.	Whether the construct is defined and delimited (D1) and whether the correlates are justified as specific to it (D2).
P2. Technological independence	—	The framework remains valid as devices and acquisition configurations change.	Nothing. This is a property of the framework, not of an implementation, and is not scored.
P3. Deployment-oriented design	D7	Operating conditions are considered from the earliest design stages.	Whether the acquisition and operating constraints of the configuration used are reported.
P4. Inference traceability	D5	Outputs remain relatable to the acquisition, representation and modeling stages that produced them.	Whether an analyzed mapping from signal to feature to output is documented.
P5. Real-world validation	D6	Evaluation is not confined to performance obtained under controlled conditions.	Which evaluation regimes are reported, and on what sample.
P6. Operational viability	D7, D8	Feasibility of use and governance of use are part of the evaluation, not addenda to it.	Whether deployment constraints (D7) and the ethical and privacy conditions of use (D8) are reported.

D3 (neuroelectric representation) and D4 (computational modeling) are the technical-rigor slots jointly constrained by P1 and P4; neither descends from a single principle.

**Table 5:** Validation evidence levels for EEG–AI systems compatible with the Fear-EEG-AI Framework. Indicators are independent, not cumulative.

ID	Evidence Type	Minimum Criterion to Be Credited	Insufficient Evidence
VM1	Retrospective/offline	Evaluation on previously acquired recordings or a public dataset, with a reported evaluation regime and metrics	Anecdotal or unreported evaluation
VM2	Cross-subject	A regime in which test data come from participants absent from training and tuning, with a declared minimum usable sample in both partitions (n≥12 participants in total and ≥2 in the held-out partition), and with the demographic composition of the sample reported for age and sex and the recruitment base declared (convenience, student, clinical or community)	Within-subject *k*-fold cross-validation, however many folds; a cross-subject partition whose sample composition is not reported
VM3	Cross-session/longitudinal	Evaluation across separate sessions, days or time points, with stability reported	Repeated blocks within a single session
VM4	Real-time	Inference computed online during acquisition, with latency or continuous-processing constraints reported	Offline analysis of data acquired in real time
VM5	Operational	Evaluation with end users in a representative environment, with operational constraints of the application context reported, and with the deployment population described and its correspondence with the intended target population stated	Controlled laboratory simulation of the application context; deployment with end users whose composition is not reported

**Table 6:** Inter-rater agreement by reporting dimension over the eight eligible studies (n=8 paired scores per dimension). Pooled over all 64 pairs: κ=0.539 (unweighted), 0.636 (linear), 0.741 (quadratic); ICC(2,1) = 0.744; ICC(2,*k*) = 0.853.

Dimension	Exact	Within One Point	Mean Abs. Dev.
$D_{1}$ Construct delimitation	50.0%	100.0%	0.500
$D_{2}$ Neuroelectric correlates	62.5%	100.0%	0.375
$D_{3}$ Neuroelectric representation	75.0%	100.0%	0.250
$D_{4}$ Computational modeling	75.0%	100.0%	0.250
$D_{5}$ Inference traceability	50.0%	87.5%	0.625
$D_{6}$ Experimental validation	50.0%	100.0%	0.500
$D_{7}$ Deployment readiness	87.5%	100.0%	0.125
$D_{8}$ Ethical and privacy aspects	87.5%	87.5%	0.250
**Pooled**	**67.2%**	**96.9%**	**0.359**

**Table 7:** Adjudicated matrix of reporting alignment, alignment index and validation-evidence profile for the eight eligible studies of the corpus. Scores are the consensus of two independent raters after adjudication; the 95% interval is that of Section 3.5 under admissible weightings. The index is not used to order individual implementations.

Evaluated Study	$D_{1}$	$D_{2}$	$D_{3}$	$D_{4}$	$D_{5}$	$D_{6}$	$D_{7}$	$D_{8}$	FAI (%)	95% CI
Alakus et al. [56]	2	2	2	3	3	2	3	1	45.0	35.8–53.3
Bălan et al. [53]	4	4	4 ^a^	4	4	2	2	1	62.5	46.0–75.3
de Man & Stassen [59]	2	2 ^b^	1	1	3	2	3	2	40.0	30.5–49.4
Ishizuka et al. [55]	2	2	2	4	3	2	3	1	47.5	36.8–59.7
Kim et al. [58]	2	3	2	1	2	2	3	2	42.5	34.1–50.3
Krishna et al. [57]	2	1	2	3	3	2	3	1	42.5	31.8–52.4
Proverbio & Cesati [54]	3	4	4	1	2	2	2	2	50.0	37.7–63.9
Turnip et al. [60]	1	2	2	2	2	2	2	2	37.5	31.9–39.9
**Mean**	2.25	2.50	2.38	2.38	2.75	2.00	2.63	1.50	45.9	

^a^ Capped at anchor 4 by rule R3, the preprocessing having been inherited from a public repository. ^b^ Capped at anchor 2 by rule R2, the inference resting on a single frontal channel without documented artifact control; the raters had split 2/3 on this cell and the adjudication resolved it under that rule. Rows are ordered alphabetically. All eight studies satisfy VM1 and none satisfies VM2–VM5, so VM-max and VM-core equal VM1 throughout and the column is omitted; Section 3.7 discusses that outcome.

## Data Availability

No new EEG data were generated. The scoring rubric, its application rules, the scoring protocol, the corpus eligibility decisions, the adjudicated score matrix, the agreement statistics, the adjudication record for every cell in which the raters differed, the weighting specifications and the results of the sensitivity analyses are provided as Supplementary Materials in the form in which they were applied. The studies scored are published sources cited in the reference list.

## Supplementary Materials

The following supporting information can be downloaded at https://www.mdpi.com/article/10.3390/s26196349/s1. The supplementary document is self-contained and comprises: S1, Scoring protocol as applied; S2, Scoring rubric and application rules as applied; S3, Corpus eligibility decisions; S4, Weight vectors of the deterministic specifications; S5, Stochastic and structural specifications; S6, Minimum usable sample for indicator VM2 (cross-subject evidence); S7, Crosswalk to the TRIPOD+AI checklist; S8, Adjudicated score matrix; S9, Inter-rater agreement; S10, Adjudication of the 21 discrepant cells; S11, Per-study profiles; and S12, Computed results.

## Abbreviations

The following abbreviations are used in this manuscript:

AI	Artificial Intelligence
AUC	Area Under the Curve
BCI	Brain-Computer Interface
CWT	Continuous Wavelet Transform
DWT	Discrete Wavelet Transform
EEG	Electroencephalography
EMG	Electromyography
EOG	Electrooculography
ERD	Event-Related Desynchronization
ERS	Event-Related Synchronization
FAI	Fear-EEG-AI Alignment Index
Fear-EEG-AI	Framework for EEG-based inference of fear-compatible neurophysiological states
ICA	Independent Component Analysis
ICC	Intraclass Correlation Coefficient
LSTM	Long Short-Term Memory
MEG	Magnetoencephalography
PFC	Prefrontal Cortex
PSD	Power Spectral Density
RNN	Recurrent Neural Network
SRL	System Readiness Level
TRL	Technology Readiness Level
VM	Validation Evidence Level

## Appendix A. FAI Scoring Rubric and Application Protocol

### Appendix A.1. Anchored Scoring Rubric

The Fear-EEG-AI Alignment Index (FAI) aggregates eight dimensions, each scored on an ordinal scale from 1 to 5. To reduce subjectivity and make scores reproducible across raters and studies, each score level is defined by an explicit anchor descriptor. Consistent with the purpose of the FAI, the rubric evaluates what a study reports and how it is justified—not the absolute scientific merit of the underlying result. A dimension is scored on the basis of the information explicitly available in the source, and the absence of reported information is scored as a deficiency rather than assumed in the study’s favor. Table A1 presents the full rubric. All eight dimensions use the same five-level scale, so $D_{\max}=5$ uniformly and the aggregation of Equation (1) is well defined without per-dimension normalization; the ordinal-versus-interval assumption that this aggregation carries is stated in Section 3.4. The anchors of D2 and D5 are stated in terms of conditions a rater can verify in the source, since these are the two dimensions whose descriptors are least determinate when expressed qualitatively. They are applied together with the rules of Appendix A.2: rule R2 for D2 and rule R4 for D5.

**Table A1:** Anchored scoring rubric for the eight dimensions of the Fear-EEG-AI Framework. Each dimension is scored from 1 (critical deficiency or absence of reported information) to 5 (exemplary and fully documented).

Dimension	Score	Anchor Descriptor
**D1. Construct delimitation**	1	No explicit definition of the target state; fear is treated as an absolute label fed to a classifier with no neuroscientific justification.
	2	The target state is named but not delimited from adjacent states (anxiety, acute stress, generic high arousal); neuroscientific grounding is generic or absent.
	3	The target state is defined and partially delimited; threat-related circuitry is mentioned, but the link between that circuitry and the proposed inference is loose or implicit.
	4	The construct is explicitly defined and delimited from adjacent emotions; relevant threat-appraisal circuitry is identified and the inference is justified with cited evidence, with minor gaps.
	5	Rigorous construct definition with an explicit emotional-confusion space; threat-appraisal circuitry is mapped to expected observable cortical effects; the inference is fully justified and appropriately hedged (“compatible-with-fear” rather than absolute detection).
**D2. Neuroelectric correlates**	1	No correlate is named, or features are purely data-driven and no physiological rationale is stated.
	2	Correlates are named; neither a physiological justification nor any stated link to the target construct is reported. This level also applies, by rule R2, whenever the inference rests on a single frontal channel and artifact control is not documented.
	3	Correlates are named with a rationale that cites the affective literature in generic terms (arousal, valence, attention) without establishing specificity to threat processing. This level also applies, by rule R2, whenever the inference depends on correlates above 30 Hz and artifact control is not documented.
	4	The rationale cites evidence on threat processing specifically; two or more complementary correlates are reported; artifact control for the bands actually used is documented. Specificity limits are not discussed.
	5	As level 4, and the report additionally states which adjacent affective states share the reported signature—coherent with the delimitation scored in D1—and the artifact susceptibility of each correlate used.
**D3. Neuroelectric representation**	1	Acquisition/preprocessing is not reported or is clearly inadequate (e.g., single channel with no artifact control).
	2	Basic acquisition is reported with minimal preprocessing; no advanced artifact handling (no ICA or validated rejection); reproducibility is doubtful.
	3	An adequate pipeline (filtering, segmentation) with some artifact control is reported; feature extraction is described but only partially standardized.
	4	A robust, well-documented pipeline with explicit artifact handling and reproducible feature extraction across the stated montage; minor omissions remain.
	5	A fully standardized, reproducible pipeline with rigorous artifact control (ICA or validated rejection), signal-quality estimation, and complete parameter reporting.
**D4. Computational modeling**	1	The model is unspecified or inappropriate for the task; the choice is not validated.
	2	A model is reported without justification; training and validation details are missing.
	3	An appropriate model with basic validation is used; performance is reported, but interpretability and efficiency are not addressed.
	4	The model choice is justified with proper training/validation, and at least one of interpretability, efficiency, or robustness is explicitly addressed.
	5	The model choice is explicitly justified against the interpretability/deployment trade-off, with strong validation and all of performance, interpretability, efficiency, and robustness addressed.
**D5. Inference traceability**	1	The output is reported as a label or score with no reference to the features that produced it.
	2	The processing pipeline is described, but no analysis relates the output to the features: no feature importance, no explicit model rules and no sensitivity of the decision are reported.
	3	The relation between features and output is inferable from the description but is not analyzed or quantified anywhere in the report.
	4	At least one of the following is reported and covers part of the pipeline: a feature-importance analysis, the explicit decision rules of the model, or a sensitivity analysis of the decision.
	5	The mapping is reported end to end, from the acquired signal through the representation to the output, together with one of the analyses required at level 4 and a statement of which stages remain unverified. By rule R4, the determinism of a transform or the structural transparency of a model class does not on its own satisfy levels 4 or 5.
**D6. Experimental validation**	1	No validation, or anecdotal evaluation with no reported metrics.
	2	Offline validation on a small or biased sample with a single evaluation regime; overfitting risk is not controlled.
	3	Adequate offline validation with proper cross-validation on a reasonably representative sample, but a single evaluation regime.
	4	Cross-subject and/or cross-session (or real-time) validation with an adequate sample and error analysis; sample-representativeness limits may remain.
	5	Progressive validation (cross-subject, cross-session, and real-time) with an adequate sample, error analysis, and evidence of temporal stability.
**D7. Deployment readiness**	1	Deployment is not considered; the setup requires heavy laboratory infrastructure with no path to transfer.
	2	A high-density/wet-electrode or externally-tethered configuration is used; portability and operational constraints are not addressed.
	3	Some deployment factors (portability or latency) are considered, but major constraints remain (complex setup, tethered computation).
	4	A portable, low-setup configuration with edge or low-latency processing addresses most operational constraints; minor gaps remain.
	5	Fully deployment-oriented: portable low-cost hardware, on-device processing, and latency, energy, continuous-operation, and usability all addressed and justified.
**D8. Ethical and privacy aspects**	1	No ethical or privacy consideration is reported.
	2	Only baseline compliance is reported (e.g., a generic informed-consent/ethics-approval statement), with nothing on data handling, scope limits, or error consequences.
	3	Baseline compliance plus basic data handling (e.g., anonymization/data protection) or an explicitly regulated governance context (e.g., a pre-approved public dataset), but no privacy-by-design.
	4	Compliance plus at least one advanced safeguard: data encryption, false-positive/negative management, or explicit delimitation of clinical/operational scope.
	5	Privacy-by-design: data minimization/on-device processing, informed consent adapted to the target population, false-positive/negative management, scope limits, and protection of vulnerable populations.

### Appendix A.2. Application Rules

The anchor descriptors of Table A1 do not by themselves determine a score without tacit judgment in four recurring situations. The following rules resolve them; they were fixed before the corpus was scored and are part of the instrument rather than commentary on it.

R1. **Evaluation regime (D6).** *k*-fold cross-validation within subject constitutes a single evaluation regime. Anchor 4 requires at least one regime that breaks subject dependence, breaks session dependence, or operates online. The number of folds does not substitute for diversity of regime.
R2. **Specificity and artifact susceptibility (D2).** Where the inference depends on correlates above 30 Hz and the study does not document control of electromyographic and ocular artifact, D2 is capped at 3. Where it depends on a single frontal channel without artifact control, D2 is capped at 2. This rule makes the scoring consistent with Section 2.2.6, which treats gamma as an optional feature conditional on signal quality: crediting high alignment to gamma-derived correlates in the absence of that control would contradict the framework the index operationalizes. The diagnostics that make such a criterion auditable, and the reason no universal threshold is proposed, are given in Section 2.2.6.
R3. **Secondary data (D3).** Where a study reuses a public repository, D3 is scored on the declared traceability of data provenance and on the additional processing the authors themselves perform and describe. D3 is capped at 4 and the cell is flagged as inherited, because the acquisition and preprocessing pipeline was not executed by the authors and signal-quality estimation for the specific use is not thereby established.
R4. **Determinism is not traceability (D5).** The deterministic character of a transform, or the structural transparency of a model class, does not contribute to D5 on its own. A score of 4 or above requires an analyzed signal–feature–model–result mapping: feature-importance analysis, explicit model rules, or sensitivity analysis of the decision. In particular, a claim that each component of an adaptive decomposition corresponds directly to a distinct physiological process is not admissible where mode mixing is known to affect that decomposition.
R5. **Source format.** The publication format and its length limit are recorded for each source. Scores are not adjusted for format, and the dependence of the instrument on reporting space is declared as a limitation rather than corrected for.
