# Supplementary material for: Fear-EEG-AI: A Conceptual and Operational Framework for Designing EEG-AI Systems for the Inference of Fear-Compatible Neurophysiological States
Source: Sensors (Basel). 2026 Oct 8;26(19):6349. doi: 10.3390/s26196349 (PMC13667737; doi:10.3390/s26196349)
Supplement: Supplementary file 1 [file sensors-26-06349-s001.zip › sensors-4564203-supplementary.pdf]

Supplementary Material

Fear-EEG-AI: A Conceptual and Operational Framework for Designing EEG–AI Systems for the Inference of Fear-Compatible Neurophysiological States

This document contains every item the manuscript cites as Supplementary Material. Section numbers below are the ones the manuscript refers to. Rubric anchors (Appendix A.1) and application rules (Appendix A.2) are reproduced here as Section S2 so that the instrument as applied can be read without the article.

S1. Scoring protocol as applied

This section reproduces the protocol under which the corpus was scored. It was written and fixed before scoring began; the manuscript states in its limitations that the ordering of these events is documented but not independently timestamped.

Object of measurement. The rubric scores *what a publication reports*, not the quality of the underlying system. A low score indicates that a methodological decision is absent from the report or insufficiently documented; it is not an appraisal of the study's validity, and the instrument does not substitute for methodological appraisal.

Eligibility. Two criteria, fixed before scoring, applied to the eleven studies retained by the seed systematic review. A study is eligible if (I1) it acquires or reuses electroencephalographic signals, since the representation dimensions are not applicable otherwise, and (I2) it is a primary study rather than a secondary synthesis. Section S3 records the decision and its basis for each of the eleven.

Admissible sources. The published article and its own supplementary material, plus the primary documentation of a reused public dataset. Information not available in those sources was treated as not reported, which is the behavior the instrument measures. Author correspondence, preprints and later publications by the same group were not admissible.

Raters and procedure. Two raters scored the eight eligible studies independently. Neither had drafted the rubric, the application rules or the protocol, and neither had access to the other's scores or to any previous scoring of these studies. Studies were presented to each rater in a different random order. Each rater recorded, for every cell, the anchor applied, the application rules invoked and the supporting passage of the source. Every disagreement was resolved by a third rater, who recorded the anchor applied and the rule invoked. Agreement statistics are computed on the scores prior to adjudication; the matrix reported in the manuscript is the adjudicated one.

Planned analysis. Fixed before scoring: inter-rater agreement per dimension and over the pooled cells (percentage exact, percentage within one point, mean absolute deviation, Cohen's kappa unweighted and under linear and quadratic weighting, and the two-way intraclass correlation coefficient for a single rater and for the mean of two); the composite index under the deterministic weighting specifications of Section S4; the stochastic and structural sensitivity analyses of Section S5; and the validation-evidence profile per study.

Limitations declared in advance. Three were stated before scoring and are reported in the manuscript. First, publication format is a confound: short-format sources have less room to document decisions and are penalized by construction. Second, the corpus derives from a search strategy anchored on one term and is not a representative sample of EEG-based threat research. Third, with eight studies no inferential analysis of association between the index and any other quantity is admissible, and none is reported.

Deviations from the protocol. None. In one cell (de Man & Stassen, D2) the anchor descriptor and rule R2 pointed to different values; the adjudicator applied the rule in preference to the descriptor, and the cell was resolved to 2.

S2. Scoring rubric and application rules as applied

The anchored rubric is Table A1 of the manuscript and is not duplicated here. The five application rules, fixed before scoring and part of the instrument rather than commentary on it, are:

R1. Evaluation regime (D6). k-fold cross-validation within subject constitutes a single evaluation regime. Anchor 4 requires at least one regime that breaks subject dependence, breaks session dependence, or operates online. The number of folds does not substitute for diversity of regime.

R2. Specificity and artifact susceptibility (D2). Where the inference depends on correlates above 30 Hz and the study does not document control of electromyographic and ocular artifact, D2 is capped at 3. Where it depends on a single frontal channel without artifact control, D2 is capped at 2. This rule makes the scoring consistent with the subsection of the manuscript on gamma-band admissibility, which treats gamma as an optional feature conditional on signal quality: crediting high alignment to gamma-derived correlates in the absence of that control would contradict the framework the index operationalizes. The diagnostics that make such a criterion auditable, and the reason no universal threshold is proposed, are given in the subsection of the manuscript on gamma-band admissibility.

R3. Secondary data (D3). Where a study reuses a public repository, D3 is scored on the declared traceability of data provenance and on the additional processing the authors themselves perform and describe. D3 is capped at 4 and the cell is flagged as inherited, because the acquisition and preprocessing pipeline was not executed by the authors and signal-quality estimation for the specific use is not thereby established.

R4. Determinism is not traceability (D5). The deterministic character of a transform, or the structural transparency of a model class, does not contribute to D5 on its own. A score of 4 or above requires an analyzed signal–feature–model–result mapping: feature-importance analysis, explicit model rules, or sensitivity analysis of the decision. In particular, a claim that each component of an adaptive decomposition corresponds directly to a distinct physiological process is not admissible where mode mixing is known to affect that decomposition.

R5. Source format. The publication format and its length limit are recorded for each source. Scores are not adjusted for format, and the dependence of the instrument on reporting space is declared as a limitation rather than corrected for.

S3. Corpus eligibility decisions

The eleven studies retained by the seed systematic review, with the decision and its basis for each. Eight were eligible and all eight were scored. The first column carries the numbering of the seed review, not of this manuscript; in this manuscript the eight eligible studies and the two excluded databases appear in the reference list under their own numbers.

Ref. in seed r eview	Study	EEG	Inference model	Type	Elig ible	Basis of the decision
[54]	Proverbio & Cesati 2024	Yes (128 channels)	Not explicit (a BCI application is mentioned)	Primary EEG study	Yes	Primary EEG study; the absence of an explicit inference model does not affect eligibility, since criterion I1 requires EEG and not an AI model.
[59	de Man & Stassen 2017	Yes (1 channel, BrainLink)	No	Primary EEG study	Yes	Primary EEG study. The seed review records no AI model for this source; it is eligible because criterion I1 requires EEG acquisition or reuse.
[53]	Bălan et al. 2019	Yes (DEAP, 32 channels)	Yes (naive Bayes, SVM, k-NN, LDA, MLP)	Primary EEG study with an AI model	Yes	Primary EEG study with an AI model.
[58]	Kim et al. 2019	Yes (8 channels)	No (ERP and statistics only)	Primary EEG study	Yes	Primary EEG study.
[57]	Krishna et al. 2019	Yes (DEAP, 32 channels)	Yes (MLP, random forest, SVM; 10-fold)	Primary EEG study with an AI model	Yes	Primary EEG study with an AI model.
[62]	Cao et al. 2014	No (audio-v ideo and facial only)	No	Audio-visual dataset construction	No	Excluded by criterion I1: no EEG. The representation dimensions are not applicable.

Ref. in seed r eview	Study	EEG	Inference model	Type	Elig ible	Basis of the decision
[63]	Sneddon et al. 2012	No (audio-v ideo and facial only)	No	Audio-visual dataset construction	No	Excluded by criterion I1: no EEG.
[56]	Alakus et al. 2020	Yes (Emotiv EPOC, 14 channels)	Yes (kNN, SVM and MLP-ANN)	Primary EEG study with an AI model	Yes	Primary EEG study with an inference model: the source reports classification of discrete-wavelet features by kNN, SVM and a multilayer perceptron under 10-fold cross-validation, with accuracy and kappa as performance criteria.
[55]	Ishizuka et al. 2024	Yes (8 channels, 500 Hz)	Yes (SVM with RBF kernel)	Primary EEG study with an AI model	Yes	Primary EEG study with an AI model.
[60]	Turnip et al. 2024	Yes (19 channels, Mitsar 202)	Yes (decision trees)	Primary EEG study with an AI model	Yes	Primary EEG study with an AI model.
[6]	Vempati et al. 2023	Varies (review of >90 studies)	Yes (reviewed)	Secondary study	No	Excluded by criterion I2: a systematic review is not an implementation that the rubric can score.

S4. Weight vectors of the deterministic specifications

Weights sum to one; the index is computed as $(\Sigma_i w_i D_i) / 5 \times 100$. S2, S3 and S4 were fixed on substantive grounds before the analysis was run.

Dimension	S1 uniform	S2 domains equal	S3 clinical-assistive	S4 lab-methodological
D1 Construct delimitation	0.1250	0.1667	0.18	0.10
D2 Neuroelectric correlates	0.1250	0.1667	0.10	0.12
D3 Neuroelectric representation	0.1250	0.1111	0.10	0.18
D4 Computational modeling	0.1250	0.1111	0.10	0.18
D5 Inference traceability	0.1250	0.1111	0.08	0.16
D6 Experimental validation	0.1250	0.1111	0.18	0.14
D7 Deployment readiness	0.1250	0.1111	0.16	0.06
D8 Ethical and privacy aspects	0.1250	0.1111	0.10	0.06

Rationale. S2 gives equal weight to each of the three evaluation domains, so the two scientific-coherence dimensions receive 1/6 each and the remaining six receive 1/9 each. S3 represents a clinical or assistive deployment, where construct delimitation (D1), validation (D6), deployment feasibility (D7) and governance (D8) carry the decision. S4 represents a methodological laboratory study, where signal representation (D3), modeling (D4) and traceability (D5) carry it.

Index values the four specifications produce (per study, and the 95% interval under S5):

Study	S1 unif.	S2 domains	S3 clinical	S4 lab	95% CI (S5)
Alakus et al. 2020	45.0	44.4	44.8	46.8	35.8 – 53.3
Bălan et al. 2019	62.5	64.4	60.4	68.4	46.0 – 75.3

Study	S1 unif.	S2 domains	S3 clinical	S4 lab	95% CI (S5)
de Man & Stassen 2017	40.0	40.0	40.8	37.2	30.5 – 49.4
Ishizuka et al. 2024	47.5	46.7	46.8	50.4	36.8 – 59.7
Kim et al. 2019	42.5	43.3	43.2	40.0	34.1 – 50.3
Krishna et al. 2019	42.5	41.1	42.8	44.4	31.8 – 52.4
Proverbio & Cesati 2024	50.0	52.2	49.6	50.4	37.7 – 63.9
Turnip et al. 2024	37.5	36.7	36.4	38.0	31.9 – 39.9

S5. Stochastic and structural specifications

- **S5** — 10,000 weight vectors drawn from a Dirichlet(α = 1) distribution over the eight-dimensional simplex, i.e. uniform over all admissible weightings. Seed 20260915.
- **S6** — eight leave-one-dimension-out variants, each with uniform weights over the remaining seven dimensions.
- **S7** — perturbation of individual rubric cells by one point within the 1–5 range, bounding the effect a single scoring disagreement can have on the index. The perturbation is of one point because that is the modal disagreement actually observed between the raters: 19 of the 21 discrepant cells differed by one point and 2 by two.

S6. Minimum usable sample for indicator VM2 (cross-subject evidence)

The threshold was set by asking what sample makes a cross-subject claim resolvable at all. At a proportion of 0.80 of participants above chance, the 95% Wilson interval for that proportion has the following half-widths (the full interval is twice as wide):

Participants	95% Wilson interval	Half-width
5	0.38 – 0.96	29.4 pp
6	0.41 – 0.96	27.6 pp
8	0.45 – 0.95	24.8 pp
10	0.49 – 0.94	22.7 pp
12	0.52 – 0.94	21.0 pp
15	0.55 – 0.93	19.1 pp
20	0.58 – 0.92	16.8 pp
30	0.63 – 0.90	13.9 pp

At n = 5 a single participant moves the observed proportion by 20 points and the interval spans almost 60, so the sample cannot distinguish a result that generalizes across participants from one that does not. The threshold is therefore set at **n ≥ 12 participants with at least two held out**, where the half-width first falls to about 21 pp. Even there the estimate is coarse, which is why VM2 is a binary indicator rather than a graded one.

S7. Crosswalk to the TRIPOD+AI checklist

Item numbers refer to the TRIPOD+AI checklist (Collins et al., *BMJ* 2024;385:e078378). The mapping is descriptive and is to the principal items rather than exhaustive at the sub-item level: it records which dimension of this framework corresponds to which checklist items, and what each dimension requires that the checklist does not. Sub-items not named here are either covered by the principal item listed for the same dimension or fall among the items declared below as having no counterpart.

Dim.	Covering items	What the checklist already requires	What the dimension adds
**D1** Construct delimitation	3a, 4, 8a	Requires the outcome to be defined and its choice justified, assuming it is ascertainable by an established method.	Whether the predicted construct is neurophysiologically identifiable at all, and whether it is delimited from adjacent affective states that share its signature. A latent affective state has no gold-standard ascertainment, so defining it is not sufficient.
**D2** Neuroelectric correlates	9a, 9b	Requires the choice and definition of predictors, including how and when measured.	Whether the chosen predictors are physiologically specific to the target construct rather than to generic arousal or attention, and whether their susceptibility to non-neural artifact is documented.
**D3** Neuroelectric representation	5a, 7, 11	Requires the data source, the preparation steps and the handling of missing data to be described.	Modality-specific adequacy of acquisition and preprocessing: montage and channel count, sampling rate, artifact identification and control, and signal-quality estimation. This dimension adopts COBIDAS MEEG and EEG-BIDS rather than replacing them.
**D4** Computational modeling	12a-12g, 13, 15, 21, 22, 23a	Covers model type and rationale, data partitioning, hyperparameter tuning, internal validation, class imbalance, model output and performance measures.	Nothing substantive. This is the dimension where the existing guidance is most complete and the framework adds least; D4 is best read as a pointer to TRIPOD+AI.
**D5** Inference traceability	22, 25	Requires the final model to be specified in enough detail to be used, and the results to be interpreted.	An analyzed mapping from signal to feature to model output: feature-importance analysis, explicit decision rules, or sensitivity of the decision. Specifying a model is not the same as tracing the physiological chain that supports its output.
**D6** Experimental validation	10, 16, 23a, 24	Requires justification of study size, identification of differences between development and evaluation data, performance estimates and any model updating.	The regime taxonomy on which EEG generalization actually fails: subject dependence, session dependence, and online operation. A k-fold split satisfying item 12a can still leave all three untested.
**D7** Deployment readiness	27a-27c	Requires the intended implementation of the model to be described: how it is to be used in practice, by whom, and with what inputs and outputs.	The interaction between consumer-grade acquisition constraints and the signal quality the inference requires: latency, power, continuous operation and montage feasibility, none of which is a prediction-model property.
**D8** Ethical and privacy aspects	14, 17, 18d-18f	Covers fairness, ethical approval, registration, and data and code sharing.	Governance specific to neural data and to affective inference, including the regulatory restriction of emotion-recognition systems in defined contexts, and the consequences of inferring a state the participant has not declared.

Items with no counterpart in this framework, declared rather than duplicated: 2 (abstract), 6a (participant eligibility), 18a (funding), 19 (patient and public involvement), 20a (participant flow in results) and 26 (limitations). These are general reporting obligations that apply to the manuscript as a whole and are met by following the checklist directly.

Note on D4. Computational modeling is covered by items 12a–12g, 13, 15, 21, 22 and 23a essentially in full. D4 is therefore a pointer to existing guidance rather than a contribution of this framework, and is reported as such.

Note on COBIDAS MEEG and EEG-BIDS. No item-level crosswalk is provided for these. Their recommendations are adopted within dimension D3 rather than restated, which is the relationship the manuscript declares in Section 1.1.

S8. Adjudicated score matrix

Scores are the consensus of two independent raters after adjudication by a third. VM1 is credited for all eight studies and VM2-VM5 for none, so VM-max and VM-core equal VM1 throughout.

Study	D1	D2	D3	D4	D5	D6	D7	D8	FAI (%)	95% CI
Alakus et al. 2020	2	2	2	3	3	2	3	1	45.0	35.8–53.3
Bălan et al. 2019	4	4	4[a]	4	4	2	2	1	62.5	46.0–75.3
de Man & Stassen 2017	2	2[b]	1	1	3	2	3	2	40.0	30.5–49.4
Ishizuka et al. 2024	2	2	2	4	3	2	3	1	47.5	36.8–59.7
Kim et al. 2019	2	3	2	1	2	2	3	2	42.5	34.1–50.3
Krishna et al. 2019	2	1	2	3	3	2	3	1	42.5	31.8–52.4
Proverbio & Cesati 2024	3	4	4	1	2	2	2	2	50.0	37.7–63.9
Turnip et al. 2024	1	2	2	2	2	2	2	2	37.5	31.9–39.9
**Mean**	2.25	2.50	2.38	2.38	2.75	2.00	2.63	1.50	45.9	

[a] D3 for Bălan et al. is capped at anchor 4 by rule R3 (preprocessing inherited from a public repository). [b] D2 for de Man & Stassen is capped at anchor 2 by rule R2 (the inference rests on a single frontal channel without documented artifact control); the raters had split 2/3 on this cell and the adjudication resolved it to 2 under that rule. Rows are ordered alphabetically. All eight studies fall in the *Insufficiently documented* band, since each records at least one dimension at the lowest anchor.

S9. Inter-rater agreement

Reporting dimensions, 64 study-by-dimension pairs: exact agreement 43/64 (67.2%); within one point 62/64 (96.9%); mean absolute deviation 0.359. Disagreements: 19 of one point, 2 of two points, none larger. Cohen's kappa pooled over all pairs: 0.539 unweighted, 0.636 linear, 0.741 quadratic. ICC(2,1) = 0.744; ICC(2,k) = 0.853.

Per-dimension kappa is not reported: with eight studies each dimension contributes only eight paired observations.

Dimension	Exact	Within one point	Mean abs. deviation
D1	50.0%	100.0%	0.500
D2	62.5%	100.0%	0.375
D3	75.0%	100.0%	0.250
D4	75.0%	100.0%	0.250
D5	50.0%	87.5%	0.625
D6	50.0%	100.0%	0.500
D7	87.5%	100.0%	0.125
D8	87.5%	87.5%	0.250

Validation axis, 40 binary decisions: exact agreement 36/40 (90.0%), pooled kappa 0.615. All four disagreements fell on VM1 (Proverbio & Cesati, Kim et al., Turnip et al., de Man & Stassen), the first rater recording No and the second Yes in each. All four were adjudicated as Yes on the same stated basis: each study performs a non-anecdotal offline evaluation on acquired recordings, with an analytical regime and reported metrics or tests, which is what VM1 requires — it does not require a machine-learning classifier. The record gives this one basis for all four cells and does not document a separate reason for each, so the first rater's grounds in any individual case cannot be recovered from it. Note that the four are not the studies without a classifier: Turnip et al. reports decision trees. VM2-VM5 were assigned identically by both raters in all eight studies.

S10. Adjudication of the 21 discrepant cells

Each disagreement was resolved by rereading the anchor descriptor and applying rules R1-R5. No disagreement was settled by averaging the two ordinal scores.

In the 43 cells on which the raters agreed exactly, both recorded the value shown in the matrix of Section S8, so no separate adjudication record exists and the two individual sets of 64 scores can be reconstructed in full from that matrix together with the table below.

One feature of the record should be read alongside it. In twenty of the 21 cells the resolved value coincides with the second rater's score, and in the remaining cell — the one rule R2 governs — with the first rater's. The record attributes this to those scores fitting the literal descriptors and the mandatory rules, which is consistent with the disagreements being concentrated in two readings the rules exclude, both of them the first rater's: scoring the lowest anchor on D5 and D6 wherever no classifier is reported, and crediting an upper anchor without the condition that anchor requires. The adjudicator was not blind to which rater had produced which matrix, so the concentration cannot be shown to be independent of that knowledge; the adjudicated matrix is therefore the output of the rules applied to two independent readings rather than a consensus between them.

Study	Dim	R1	R2	Adj.	Basis of adjudication
Proverbio & Cesati 2024	D5	1	2	2	The signal to ERP to source-reconstruction pipeline is described, but there is no feature-to-output analysis sufficient for anchors 4-5. Anchor 1 would be too low because an explicit processing chain is present.
Proverbio & Cesati 2024	D6	1	2	2	A formal offline evaluation with statistical tests and metrics exists, so anchor 1 (absent or anecdotal validation) does not apply. No cross-subject, cross-session or online regime is documented.
Kim et al. 2019	D5	1	2	2	The spectral processing is described, but there is no traceability analysis of a predictive decision.
Kim et al. 2019	D6	1	2	2	A quantitative offline evaluation by ANOVA is present and is not anecdotal, although no classifier and no independent validation exist.
Krishna et al. 2019	D1	1	2	2	Fear appears as the target state but is not delimited against anxiety, stress or arousal, which matches anchor 2 literally.
Krishna et al. 2019	D3	1	2	2	Acquisition, sampling and filtering are reported, but artifact control is minimal and unvalidated. Anchor 1 does not apply because the basic pipeline is described.
Alakus et al. 2020	D2	3	2	2	Beta and gamma are named together with their utility for emotion recognition, but no sufficiently specific physiological justification for threat or fear is documented. Rule R2 additionally prevents overvaluing gamma without specific artifact control.
Turnip et al. 2024	D3	3	2	2	Multichannel acquisition, filtering and segmentation are present, but no validated EOG, ICA or rejection procedure is reported. Anchor 3 requires some artifact control, which is not demonstrated.
Turnip et al. 2024	D5	3	2	2	Explaining entropy and information gain in the trees is not equivalent to showing the rules of the trained tree or a feature-to-decision analysis. Rule R4 prevents raising the score for structural transparency alone.
Ishizuka et al. 2024	D1	1	2	2	Fear is named as a class but is not defined or delimited neurobiologically against adjacent states.
Ishizuka et al. 2024	D4	3	4	4	The SVM-RBF choice is justified by generalization and evaluated in subject-dependent and subject-independent regimes, and EMD, STFT and DWT are compared, addressing robustness and generalization.
Ishizuka et al. 2024	D6	3	2	2	The leave-one-subject-out regime breaks subject dependence, but only five usable participants remain; the sample cannot be considered adequate for anchors 3-4.

Study	Dim	R1	R2	Adj.	Basis of adjudication
Ishizuka et al. 2024	D7	2	3	3	The article explicitly considers real-time operation and uses eight electrodes, but does not demonstrate latency, on-device processing, energy use or continuous operation, which anchor 4 requires.
Bălan et al. 2019	D1	3	4	4	The study defines fear through low valence, high arousal and low dominance, differentiates it from anger, and links the inference to cited threat circuits.
Bălan et al. 2019	D2	3	4	4	Several complementary correlates are reported with fear- and anxiety-specific literature and inherited EOG control from the source dataset; anchor 5 is not reached because specificity limits are not discussed systematically.
Bălan et al. 2019	D4	3	4	4	Multiple models are compared, with training and validation, regularization and feature selection documented, and overfitting addressed. Experimental validation is assessed separately and remains low.
Bălan et al. 2019	D8	3	1	1	The article reports no ethical approval, consent, anonymization or privacy provision. Use of a public dataset does not license inferring external compliance under the admissible sources of the protocol.
de Man & Stassen 2017	D1	3	2	2	Amygdala and threat circuitry are discussed, but an explicit definition and delimitation of the construct, required by anchor 3, is absent.
de Man & Stassen 2017	D2	2	3	2	Correlates and an affective, fear-related rationale exist, which would support anchor 3, but rule R2 does not permit it here: the inference rests on a single dry frontal channel without documented artifact control, and R2 caps that case at anchor 2. The adjudicator applied the rule in preference to the descriptor and resolved the split to 2.
de Man & Stassen 2017	D5	1	3	3	The chain from signal to peaks and ratios to subjective fear is inferable and partly quantified, although no feature importance, model rules or sensitivity analysis is present for anchor 4.
de Man & Stassen 2017	D6	1	2	2	A formal offline evaluation with t-tests and comparison against subjective feedback is present and is not anecdotal, but it neither breaks subject or session dependence nor operates online.

S11. Per-study profiles

The dimensional profile of each eligible study, with the acquisition setup and the inference model as reported, the cells that required adjudication, and the application rule invoked where one applies. Scores are the adjudicated values of Section S8. The per-cell basis for the adjudicated cells is in Section S10, which also states how the two individual sets of 64 scores can be reconstructed.

Study	EEG	Inference model	Profile D1-D8	Index (%)	95% CI
Alakus et al. 2020	Yes (Emotiv EPOC, 14 channels)	Yes (kNN, SVM and MLP-ANN)	2 2 2 3 3 2 3 1	45.0	35.8-53.3
Bălan et al. 2019	Yes (DEAP, 32 channels)	Yes (naive Bayes, SVM, k-NN, LDA, MLP)	4 4 4 4 4 2 2 1	62.5	46.0-75.3
de Man & Stassen 2017	Yes (1 channel, BrainLink)	No	2 2 1 1 3 2 3 2	40.0	30.5-49.4
Ishizuka et al. 2024	Yes (8 channels, 500 Hz)	Yes (SVM with RBF kernel)	2 2 2 4 3 2 3 1	47.5	36.8-59.7
Kim et al. 2019	Yes (8 channels)	No (ERP and statistics only)	2 3 2 1 2 2 3 2	42.5	34.1-50.3
Krishna et al. 2019	Yes (DEAP, 32 channels)	Yes (MLP, random forest, SVM; 10-fold)	2 1 2 3 3 2 3 1	42.5	31.8-52.4
Proverbio & Cesati 2024	Yes (128 channels)	Not explicit (a BCI application is mentioned)	3 4 4 1 2 2 2 2	50.0	37.7-63.9

Study	EEG	Inference model	Profile D1-D8	Index (%)	95% CI
Turnip et al. 2024	Yes (19 channels, Mitsar 202)	Yes (decision trees)	1 2 2 2 2 2 2 2	37.5	31.9-39.9

Study	Lowest anchor at	Cells adjudicated	Dimensions adjudicated	Rule applied	Evidence profile
Alakus et al. 2020	D8	1	D2	D2 (R2)	VM1 only
Bălan et al. 2019	D8	4	D1, D2, D4, D8	D3 (R3)	VM1 only
de Man & Stassen 2017	D3, D4	4	D1, D2, D5, D6	D2 (R2)	VM1 only
Ishizuka et al. 2024	D8	4	D1, D4, D6, D7	none	VM1 only
Kim et al. 2019	D4	2	D5, D6	none	VM1 only
Krishna et al. 2019	D2, D8	2	D1, D3	none	VM1 only
Proverbio & Cesati 2024	D4	2	D5, D6	none	VM1 only
Turnip et al. 2024	D1	2	D3, D5	D5 (R4)	VM1 only

Every study records at least one dimension at the lowest anchor, which places the whole corpus in the lowest interpretive band irrespective of index value. All eight satisfy VM1 and none satisfies VM2 to VM5, so VM-max and VM-core equal VM1 throughout.

S12. Computed results

Rank correlation with the equal-weight specification across the S5 draws: median $\rho = 0.85$, 95% range 0.37 to 0.99. The complete equal-weight ordering is reproduced in 10.1% of draws; orderings are compared on the 27 of 28 study pairs that the equal-weight specification orders strictly, the single tied pair being excluded because any convention for breaking it would be counted as a reversal. Mean width of the 95% interval: 20.0 points. Largest leave-one-dimension-out shift: 6.1 points relative to the full specification, reversing a strictly ordered pair in 3 of the 8 variants. Effect of one rubric point on the index: 2.5 points. Of the 56 ordered pairs, twelve reach the 95% dominance threshold and exactly one also satisfies the non-overlapping-interval criterion, so exactly one may be asserted: Bălan et al. 2019 over Turnip et al. 2024, dominant in 99.6% of the draws and separated by 25.0 index points. The full matrix follows and is plotted in panel b of Figure 1.

Pairwise order stability (percentage of the 10,000 S5 draws in which the study of each row scores above the study of each column; the single assertable pair is the one reaching 99.6):

Row > column	Alak.	Bălan	de Man	Ishi.	Kim	Krish.	Prov.	Turn.
Alakus et al. 2020	—	1	83	0	64	100	30	94
Bălan et al. 2019	99	—	98	98	98	99	96	99.6
de Man & Stassen 2017	17	2	—	13	25	36	13	69
Ishizuka et al. 2024	100	2	87	—	72	100	39	96
Kim et al. 2019	36	2	75	28	—	50	9	88
Krishna et al. 2019	0	1	64	0	50	—	24	81
Proverbio & Cesati 2024	70	4	87	61	91	76	—	96
Turnip et al. 2024	6	0	31	4	12	19	4	—

Every quantity reported in the manuscript and in this document derives from the adjudicated matrix of Section S8 and the weighting specifications of Sections S4 and S5. The Dirichlet draws use a fixed random seed, so the stochastic quantities are reproducible from the matrix and the specifications alone.
